# Mutual action by Gγ and Gβ for optimal activation of GIRK channels in a channel subunit-specific manner

**DOI:** 10.1038/s41598-018-36833-y

**Published:** 2019-01-24

**Authors:** Galit Tabak, Tal Keren-Raifman, Uri Kahanovitch, Nathan Dascal

**Affiliations:** 1 Department of Physiology and Pharmacology and Sagol School of Neuroscience, Sackler School of Medicine, Tel Aviv University, Tel Aviv, 69978 Israel; 2 Present Address: Virginia Tech School of Neuroscience, Blacksburg, VA 24061 USA

## Abstract

The tetrameric G protein-gated K^+^ channels (GIRKs) mediate inhibitory effects of neurotransmitters that activate G_i/o_-coupled receptors. GIRKs are activated by binding of the Gβγ dimer, via contacts with Gβ. Gγ underlies membrane targeting of Gβγ, but has not been implicated in channel gating. We observed that, in *Xenopus* oocytes, expression of Gγ alone activated homotetrameric GIRK1* and heterotetrameric GIRK1/3 channels, without affecting the surface expression of GIRK or Gβ. Gγ and Gβ acted interdependently: the effect of Gγ required the presence of ambient Gβ and was enhanced by low doses of coexpressed Gβ, whereas excess of either Gβ or Gγ imparted suboptimal activation, possibly by sequestering the other subunit “away” from the channel. The unique distal C-terminus of GIRK1, G1-dCT, was important but insufficient for Gγ action. Notably, GIRK2 and GIRK1/2 were not activated by Gγ. Our results suggest that Gγ regulates GIRK1* and GIRK1/3 channel’s gating, aiding Gβ to trigger the channel’s opening. We hypothesize that Gγ helps to relax the inhibitory effect of a gating element (“lock”) encompassed, in part, by the G1-dCT; GIRK2 acts to occlude the effect of Gγ, either by setting in motion the same mechanism as Gγ, or by triggering an opposing gating effect.

## Introduction

G protein-gated inwardly rectifying K^+^ channels (GIRK or Kir3) are a subfamily of tetrameric inwardly rectifying K^+^ channels, with 4 genes encoding 4 GIRK subunits (GIRK1–4) in mammals^1–3^. GIRKs mediate inhibitory actions of neurotransmitters that activate G protein-coupled receptors (GPCRs). GIRKs regulate neuronal excitability and are associated with a large number of neurological disorders and alcohol and drug addiction^4–6^. GIRK1, GIRK2 and GIRK3 are widely expressed in the brain, showing overlapping but distinct distribution patterns in brain structures and within neurons^4,7–9^. While GIRK1/2 is considered as most abundant brain channel, GIRK1/3 is also ubiquitous, and GIRK2 homotetramers abound in the substantia nigra^10–14^. GIRK1 and GIRK3 cannot form homotetramers, but a pore mutation in GIRK1, F137S, allows its expression as a homotetramer, denoted as GIRK1*, which is instrumental for structure-function studies^15–18^.

In response to neurotransmitters, following the GPCR-catalyzed separation of Gβγ from Gα_i/o_, GIRKs are activated by direct binding of up to four Gβγ subunits^19–28^. In addition to this GPCR-evoked activity (I_evoked_), GIRKs also show basal activity (I_basal_) that varies considerably between channels of different subunit combinations (reviewed in ref.^29^). The complex, subunit-dependent interrelationships of GIRKs with G proteins are still incompletely understood.

GIRK1 contains a 121 amino acid-long distal C-terminus (G1-dCT) that endows GIRK1-containing channels with unique characteristics. This labile (and absent form crystal structures) protein segment does not strongly bind Gβγ but it imparts high functional activity upon GIRK1-containing channels^30,31^ and high-affinity binding (“anchoring”) of Gβγ to the full cytosolic domain of GIRK1^18,32–34^. This is manifested in the recruitment of Gβγ – but not Gα – to the vicinity of these channels and high I_basal_ of GIRK1-containing channels^18,34^. G1-dCT may also carry out an additional function: it appears to contain an inhibitory element (“lock”) that reduces the extent of activation by Gβγ^18,34–36^.

Mutagenesis, structural and NMR studies indicate a major role for Gβ in Gβγ interaction with, and activation of GIRKs^26,27,37–40^. The Gγ is thought to be primarily responsible for membrane targeting and attachment of the Gβγ dimer, through C-terminal prenylation of Gγ^41–44^. Gβγ containing a non-prenylated mutant of Gγ does not activate GIRKs, presumably because of deficient PM targeting^45,46^. It is not known if Gγ plays any role in GIRK gating, besides membrane targeting. A role for Gγ in interactions and functional effects of Gβγ on several effectors has been proposed, among them adenylyl cyclase (AC) and phospholipase Cβ (PLCβ)^47–52^. A recent study has localized two key regions in the N-terminus of Gγ subunit that may contribute to high-affinity binding of Gβ_1_γ_2_ to a ternary SNARE complex^53^, suggesting that Gγ may contribute to interaction with effectors through mechanisms besides prenylation. Kawano *et al*.^54^ observed that C terminal mutants of Gβ, which do not bind Gγ, are still able to associate with GIRK1 and GIRK2 in a co-immunoprecipitation assay, but cannot activate the GIRK1/2 channel. The authors proposed that Gγ plays a more important role in GIRK gating besides aiding in membrane insertion of the Gβγ dimer^54^; however, since Gβ cannot properly fold and reach the membrane without Gγ (see Discussion), the interpretation of these results is not unequivocal.

Here we report that expression of Gγ alone can activate GIRK channels in *Xenopus* oocytes. The activation is subunit-specific: GIRK1* and GIRK1/3 are activated, GIRK2 and GIRK1/2 are not. Unlike the expressed Gβγ, which enhances I_basal_ but diminishes the agonist-evoked current, I_evoked_, Gγ increases I_basal_ but does not reduce, and under certain conditions even increases, I_evoked_. Activation by Gγ requires the presence of endogenous (ambient) Gβγ and shows a complex stoichiometric relationship with coexpressed Gβ, suggesting that Gγ acts as a “helper” for Gβγ in opening the channel, possibly by removing an inhibitory constraint imposed by the “lock” present in the GIRK1 subunit.

## Results

### Gγ enhances GIRK1* basal and evoked currents

**Figure 1:**
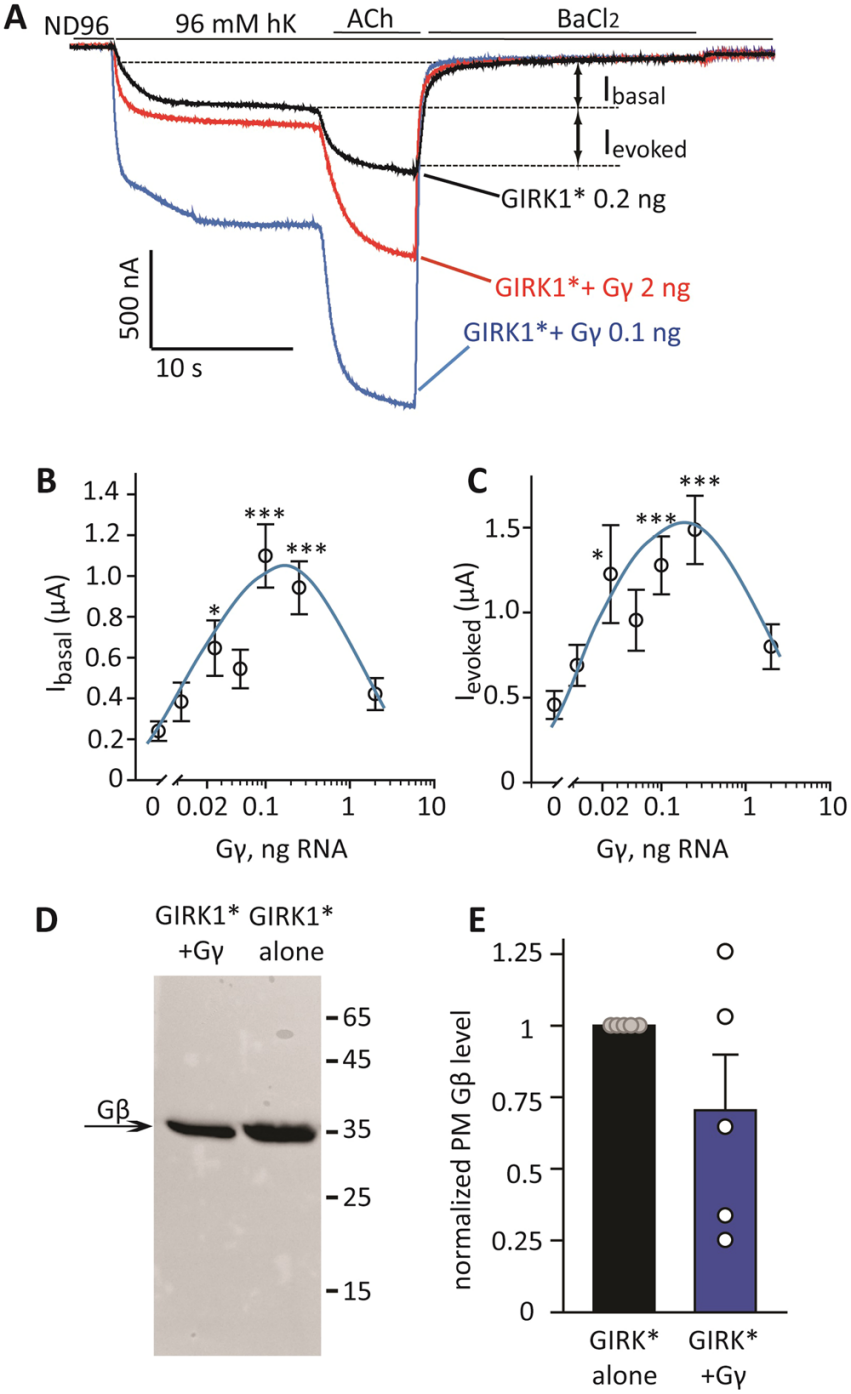
Expression of Gγ increases I_basal_ and I_evoked_ of GIRK1 without increasing the surface level of Gβ. (**A**) Representative records of GIRK currents showing that expression of Gγ (0.2 and 2 ng RNA/oocyte) increases I_basal_ and I_evoked_ of GIRK1*. GIRK1* was expressed at 0.2 ng RNA/oocyte together with m2R, 1 ng RNA/oocyte. Currents were first measured in a low-K+ solution (ND96) which was switched to the high K+ solution (hK, 96 or 24 mM K^+^, see Methods) resulting in an inward basal current, I_hK_. Then the oocyte was perfused with hK solution containing 10 µM ACh, to produce I_evoked_. At the end, 5 mM BaCl_2_ was added to the solution to block GIRK currents and to reveal the residual non-GIRK current, I_residual_. I_basal_ is defined as I_hK_-I_residual_, I_evoked_ as the net additional inward current evoked by ACh. Here I_basal_ and I_evoked_ are shown graphically for the control (GIRK1*) record. In Gγ- or Gβγ-expressing oocytes, the basal current is termed I_γ_ or I_βγ_, respectively, and defined as I_hK_-I_residual_. (**B**,**C**) Dose-dependence of the Gγ effect on I_basal_ and I_evoked_. Increasing doses of Gγ RNA were injected, together with fixed amounts of RNAs of GIRK1* and m2R (same experiment as in A). Each point shown mean ± SEM, n = 9 to 16 cells, N = 1 experiment. *p < 0.05; **p < 0.01, ***p < 0.001. (**D**) Expression of Gγ does not alter the levels of Gβ attached to the PM. The image shows Western blot of manually separated PMs (equal amounts of oocytes were used for each lane; here 25 oocytes/lane). The image was cropped from a larger one shown in Supplementary Fig. S1. Oocytes were injected with 0.2 ng of GIRK1* RNA, with or without 0.2 ng Gγ RNA. (**E**) Summary of 5 experiments (N = 5) of the kind shown in (**D**). In each experiment, the Gβ signal measured from the lane of Gγ-containing oocytes was normalized to Gβ signal of control oocytes expressing GIRK1* only. Bars represent mean ± SEM, circles show the results of individual experiments. There was no statistical difference in Gβ level with or without coexpressed Gγ, p = 0.167.

In preliminary experiments, we serendipitously discovered that heterologous expression of Gγ_2_, without Gβ, activated the GIRK1* channel. To define the role of Gγ in GIRK1* regulation, we used two-electrode voltage clamp with standard protocols^18^ to measure whole-cell GIRK currents in *Xenopus* oocytes (Fig. 1A). At a holding potential of −80 mV, shift from the low-K^+^ ND96 solution (2 mM K^+^) to a high-K^+^ (24 or 96 mM K^+^) solution induced an inward current. This current is due mostly to the basal activity of the expressed GIRK channels; the endogenous K^+^ currents in *Xenopus* oocytes are very small under these conditions^55,56^. Then the oocyte was perfused with HK solution containing 10 µM acetylcholine (ACh) which produced an evoked current, I_evoked_, due to the activation of coexpressed muscarinic m2 receptor, m2R. Full block of GIRK channels by 5 mM Ba^2+^ applied at the end of the protocol allowed calculation of net GIRK basal current, I_basal_ (Fig. 1A).

**Figure 2:**
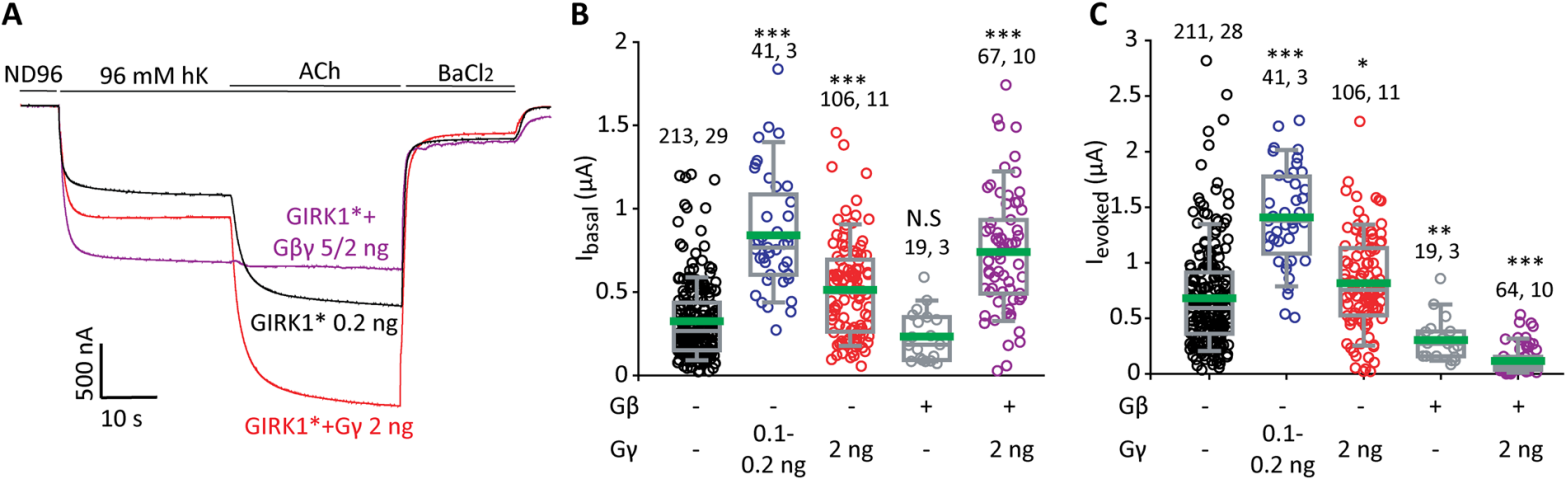
The effect of Gγ is different from the effect of coexpression of Gβγ. (**A**) Representative currents in oocytes injected with the indicated concentrations of RNAs: GIRK1*, GIRK1* with Gγ, and GIRK1* with Gβγ. m2R was coexpressed in all cases. Gγ enhances both I_basal_ and I_evoked_, whereas Gβγ enhances I_basal_ but abolishes I_evoked_. (**B**,**C**) Summary of the effects of expression of Gβ, Gγ and Gβγ on I_basal_ and on I_evoked_, respectively, of GIRK1*. The RNA concentration of GIRK1* was 0.2 ng/oocyte, RNA concentrations of Gβ and Gγ are indicated below the X axis. Data with 0.1 and 0.2 ng Gγ RNA were pulled because they produced very similar effects. The green line within the boxes shows the mean of data. Numbers at the top are n, N (number of cells, number of experiments). Statistical analysis: one-way ANOVA on ranks followed by Dunn’s test. *p < 0.05, **p < 0.01, ***p < 0.001, N.S = not significant, relative to GIRK1* alone.

Throughout this study, we used the ubiquitous Gβ_1_ and Gγ_2_ (Gβ and Gγ in the following), which are the most abundant, PM-associated neuronal Gβ and Gγ species^57^. We first studied the effect of a range of doses of Gγ RNA (“titration” of Gγ expression). With GIRK1* expressed at 0.2 ng RNA/oocyte, Gγ increased I_basal_ of GIRK1* up to 4-fold and I_evoked_ up to 3-fold (Fig. 1A–C). Interestingly, for both basal and evoked currents, the dose-response curve was bell-shaped (Fig. 1B,C). Maximal increase in I_basal_ of GIRK1* was usually obtained at relatively low doses of Gγ RNA, 0.1–0.2 ng/oocyte (2.94 ± 0.26 fold, n = 41 oocytes, N = 3 experiments). A higher dose of Gγ_2_, 1–2 ng RNA per oocyte (typically used in experiments with GIRK), usually had a milder effect (1.79 ± 0.1 fold increase in I_basal_, n = 106, N = 11; see also summary in Fig. 2B).

Gβγ is a dimer in which the partner proteins enhance each other’s stability in the cell^58,59^; however, there is evidence for stable expression of Gγ derivates, such as GFP-Gγ heterologously expressed in Dictyostelium discoideum^60^. We first addressed the possibility that heterologously expressed Gγ activates GIRK1* by recruiting the endogenous Gβ to the PM and increasing the total surface Gβγ level. We examined whether expression of Gγ affects the level of endogenous PM-attached Gβ using a method uniquely applicable to *Xenopus* oocytes, in which plasma membranes, together with the surrounding extracellular matrix (vitelline membrane), are manually separated from the rest of the oocyte^61,62^. Fig. 1D shows a Western blot of isolated PMs from oocytes injected with RNA of GIRK1* alone or together with 0.2 ng Gγ RNA. Coexpression of Gγ did not significantly change the amount of endogenous Gβ in the PM. On average, the PM level of Gβ was 70 ± 19% of control when 0.2 ng Gγ was coexpressed of (Fig. 1E, n = 5; p = 0.17). These results indicate that expression of Gγ at doses that cause maximal activation of GIRK1* is not associated with a significant recruitment of endogenous Gβ (although we cannot rule out subtle changes in PM Gβ levels which may not be detected by Western blotting).

It has been previously shown for GIRK1/4, GIRK1/2 and GIRK1* that expression of Gβγ increases I_basal_ but suppresses I_evoked_^16,18,21^. In contrast, Gγ expressed alone significantly increased both I_basal_ and I_evoked_ of GIRK1*, with a maximal effect at 0.1–0.2 ng RNA/oocyte (Fig. 2). Figure 2B,C summarizes a large amount of similar experiments that demonstrated a significant increase in both I_basal_ and I_evoked_ of GIRK1 by Gγ, with a maximal effect at 0.1–0.2 ng RNA/oocyte. Gβ expressed alone had no effect on I_basal_ but reproducibly decreased the evoked current. This is another indication that GIRK1* activation by Gγ is not due to recruitment of Gβ by Gγ.

### Gγ and various Gγ-based constructs increase GIRK1* currents but not GIRK1* surface density

**Figure 3:**
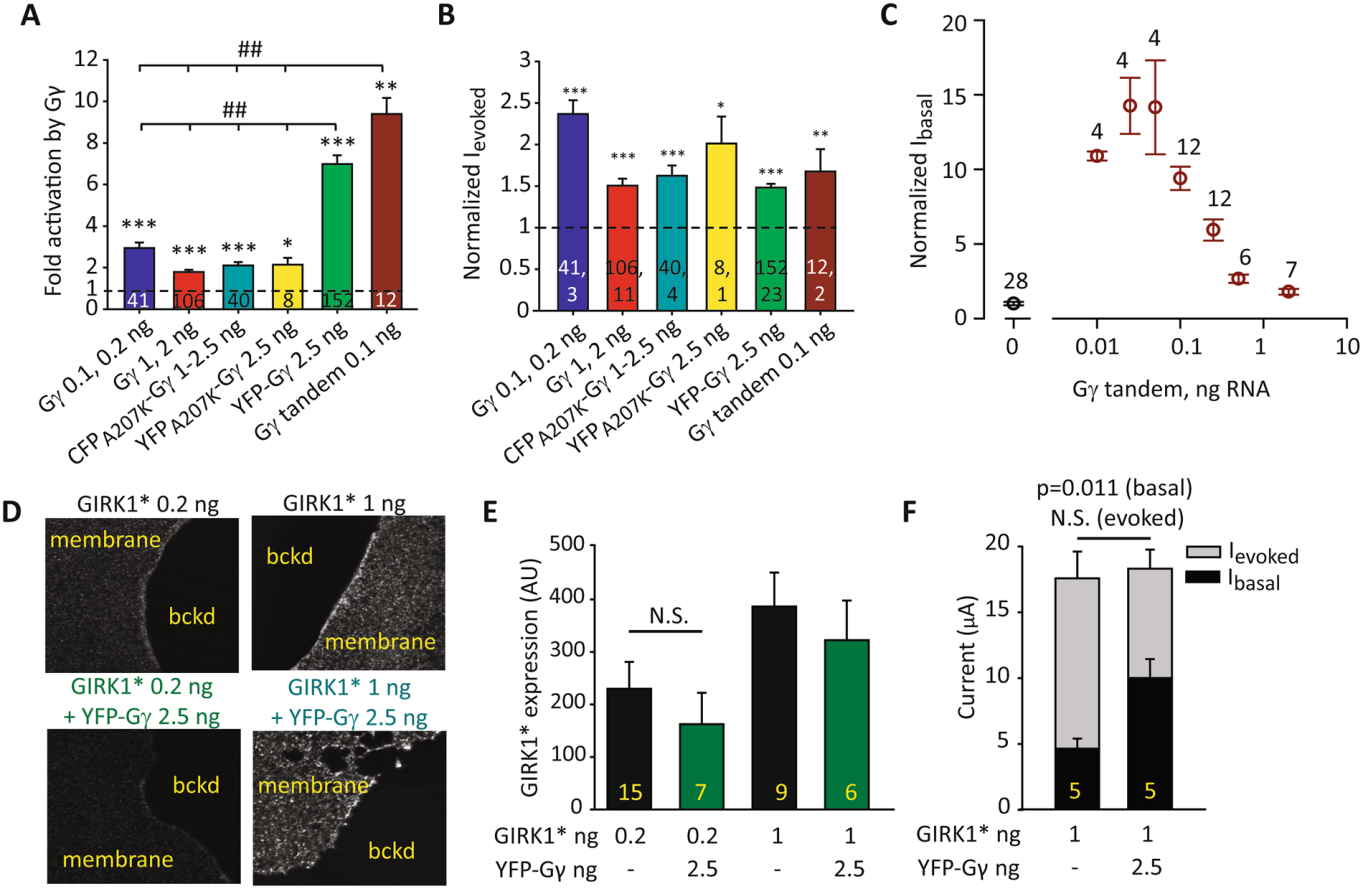
Gγ and Gγ-based constructs increase GIRK1* currents but not expression in PM. (**A**) Summary of GIRK1* activation by Gγ, expressed as fold activation, for the different Gγ constructs. Fold activation in all groups, including the control group, was calculated for each oocyte as the GIRK current in this oocyte, divided by the average current of the control group. For Gγ and CFP_A207K_-Gγ, data obtained with 2 to 3 RNA doses that produced similar effects were pooled as indicated. Statistical analysis was performed using t-test for each Gγ construct vs. control from the same experiments. * = < 0.05, ** < 0.01, *** < 0.001 in comparison to the control group, GIRK1* alone. In addition, the extent of activation was compared among all test groups using one-way ANOVA; ##, p < 0.01. (**B**) Summary of the effects of Gγ constructs on I_evoked_. Currents were normalized as follows: for each Gγ construct, in each oocyte, including the oocytes of the control group (channel alone) the value of I_evoked_ was divided by the average I_evoked_ of the control group. Statistical analysis was performed using t-test, as in A. Numbers within columns show n, N (same for **A** and **B**). (**C**) Titrated expression of Gγ tandem reveals a bell-shaped curve of dose-dependent activation of GIRK1* (0.2 ng RNA). Summary of N = 2 experiments. (**D**) Representative images of giant PM patches of oocytes expressing GIRK1* 0.2 ng or GIRK1* 1 ng alone (top) or with YFP-Gγ 2.5 ng (bottom). N = 1 experiment. PM patches were stained with an antibody against GIRK1. Membranes are seen as brighter-colored areas, background is black. (**E**) Summary of GIRK1* expression at 0.2 and 1 ng with YFP-Gγ 2.5 ng. Statistical analysis was performed by t-test. *p < 0.05, N.S., not statistically significant. A.U. - arbitrary units. (**F**) Summary of I_basal_ and I_evoked_ from the experiment in D and E, with oocytes injected with 1 ng GIRK1* RNA. The increase in I_basal_ in the presence of YFP-Gγ was significant (p = 0.011), whereas I_evoked_ was not changed significantly (p = 0.101). Statistics: t-test on raw data.

Fluorescently (YFP or CFP)-labeled Gγ constructs are often used instead of wild-type (WT) Gγ, e.g. for imaging. We tested how such Gγ-based protein constructs affect GIRK1* currents. We tested Gγ, YFP-Gγ, YFP_A207K_-Gγ (the A207K mutation prevents dimerization of YFP or CFP^63^) and CFP_A207K_-Gγ. Expression of all Gγ constructs caused a significant increase in GIRK1*’s I_basal_ and I_evoked_ (Fig. 3A,B). Effect on I_basal_ was quantified as fold increase in I_basal_. CFP_A207K_ and YFP_A207K_-tagged Gγ increased I_basal_ similarly to WT Gγ. Interestingly, the YFP-Gγ lacking the A207K mutation caused the strongest activation of GIRK1*, ~7 fold (Fig. 3A). To address the possibility that dimerization of this YFP-fused construct somehow contributes to increased potency of Gγ activation of GIRK1*, we generated a Gγ concatemeric construct (Gγ tandem) consisting of two Gγ subunits joined head-to-tail. Coexpression of the Gγ tandem strongly activated GIRK1*, similarly to YFP-Gγ (Fig. 3A,C); the dose dependency on RNA dose was bell-shaped, like in the WT Gγ (Fig. 3C). These results indicate that formation of dimers enhances the potency of Gγ. Interestingly, I_evoked_ was similarly, mildly potentiated by all Gγ constructs tested (Fig. 3B), underscoring the complexity of underlying mechanisms(s). In the following we routinely used YFP-Gγ and Gγ tandem, which produce a better channel activation than the WT Gγ, and YFP tag allows measuring Gγ expression if needed. Key experiments have been repeated with WT Gγ to verify the authenticity of the observed phenomena.

Next, we set to test whether YFP-Gγ recruits GIRK1* to the PM (which could cause an increase in the whole-cell GIRK current). Oocytes were injected with two concentrations of RNA GIRK1*, 0.2 and 1 ng, with or without YFP-Gγ (2.5 ng RNA). Giant excised PM patches were prepared from the oocytes, stained with an antibody against GIRK1, and the expression was measured using a confocal microscope (Fig. 3D). Coexpression of YFP-Gγ did not increase the level of GIRK1* in the PM (Fig. 3D,E), whereas GIRK1* basal currents measured in the oocytes of the same batch were increased (Fig. 3F). We conclude that the increase of GIRK1* current, caused by coexpressed of Gγ, is not due to an increase in the surface level of GIRK1* channels.

Interestingly, activation of GIRK1* (fold increase in I_basal_) by both YFP-Gγ and YFP-Gβγ was milder for higher expression levels of GIRK1* (1–2 ng RNA) than for the lower expression level (0.2 ng RNA; Supplementary Fig. S2). The YFP-Gγ - induced increase in I_evoked_ was 1.48 ± 0.05 fold (n = 152; Figs 3B, S2) for low GIRK1* expression levels, and no increase in I_evoked_ was observed with high GIRK1* levels (0.95 ± 0.08, n = 34; Figs 3F, S2). Poor activation by GPCR agonists and Gβγ at high levels of channel expression has been reported previously for GIRK1*^18^ and GIRK1/2^64^. In GIRK1/2, this phenomenon has been attributed to recruitment of free Gβγ by the channel, which results in increased basal activity and correspondingly reduced evoked responses^34,62^. We assume that a similar process may take place in GIRK1* which also recruits Gβγ^34^, but have not further pursued this subject here.

### Effect of Gγ on I_basal_ requires ambient Gβ

**Figure 4:**
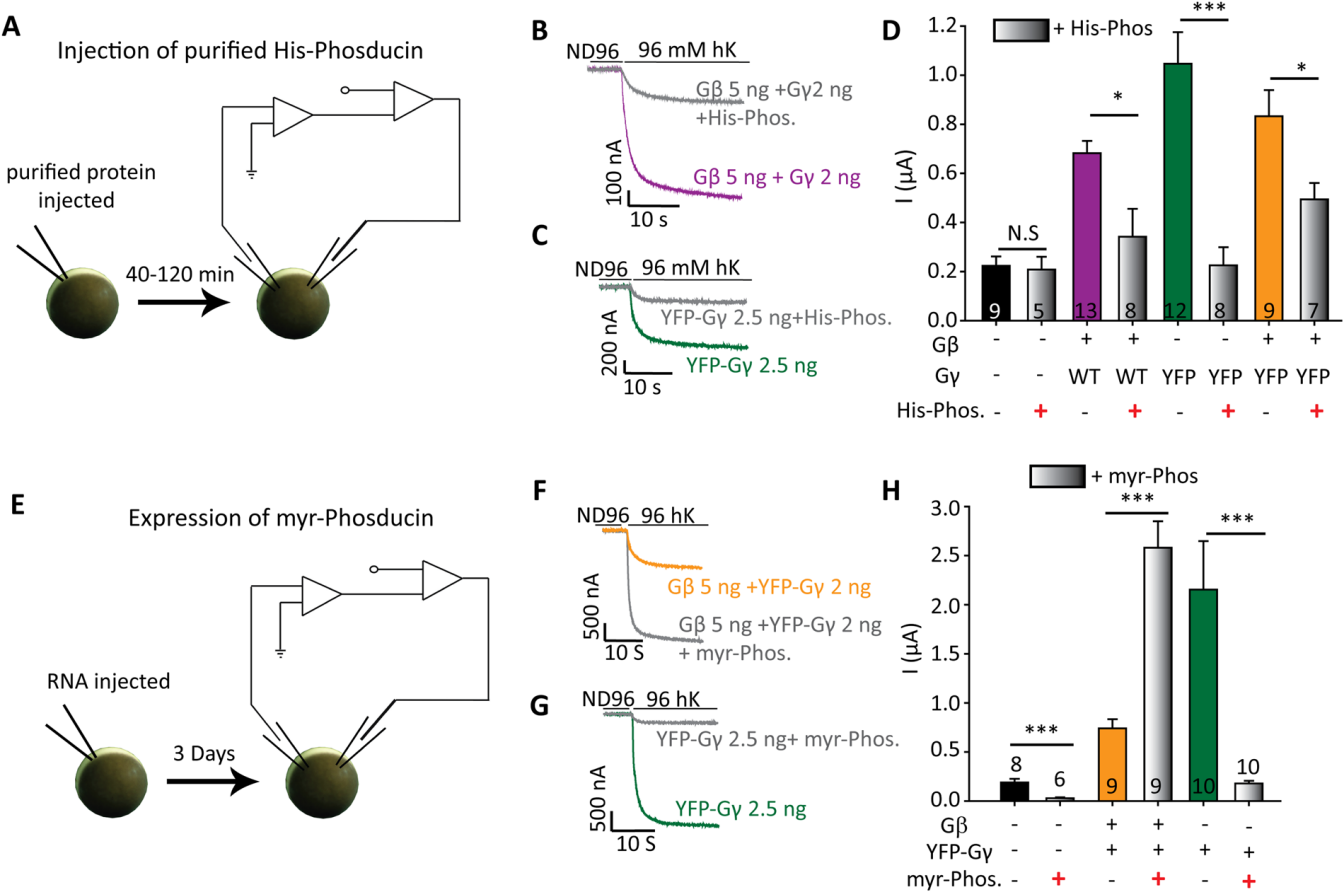
Activation of GIRK1* by Gγ requires ambient Gβ. **(A**–**D**) Injection of purified His-phosducin (His-Phos) inhibits activation of GIRK1* by Gγ. (**A**) Scheme of the experiment. At the day of the experiment, His-phosducin protein was injected into the oocytes to a final concentration of ~23 µM. After 40–50 min, currents were measured using the standard protocol. (**B**,**C**) Examples of GIRK1* currents. Oocytes were injected with GIRK1* 0.2 ng RNA/oocyte, Gβ 5 ng, YFP-Gγ 2.5 ng or Gγ 2 ng. Numbers of cells tested (n) are shown within bars; N = 1 experiment. (**D**) Summary of the experiment; His-phosducin significantly attenuates activation by Gγ and Gβγ. In this experiment, net GIRK current was calculated by subtracting average inward current measured in naïve oocytes in hK solution. Statistical analysis: t-test for each pair, with and without His-phosducin. *p < 0.05, **p < 0.01, N.S - not statistically significant. (**E–H**) Coexpression of myr-phosducin abolishes activation of GIRK1* by YFP-Gγ. (**E**) Scheme of the experiment. Myr-phosducin RNA (5 ng) was injected into the oocytes three days before the experiment, together with GIRK1* (0.2 ng RNA) and other indicated RNAs. (**F,G**) Examples of GIRK1* currents. (**H**) Summary of the results. Expression of myr-phosducin blocked the activation by YFP-Gγ but, apparently paradoxically, enhanced activation caused by Gβγ. Numbers of cells tested (n) are shown; N = 1 experiment. Statistical analysis was performed by using t-test for each pair, with and without myr-phosducin, ***p < 0.001.

Since Gβ is considered as the main GIRK-interacting partner and activating moiety, we sought to investigate the possible involvement of Gβ in Gγ-induced activation of GIRK1*. To this end, we used phosducin, a Gβγ-binding protein which is widely used as a Gβγ “scavenger”^65–67^. Phosducin interacts with Gβγ via contacts mainly in Gβ subunit^68,69^, therefore it is not expected to sequester any Gγ that is not associated with Gβ. We purified His-tagged phosducin (His-phosducin) and verified that it binds Gβγ (Supplementary Fig. S3A). We injected His-phosducin into the oocytes to a final concentration of ~23 µM within the cell, at least 40–50 minutes before measuring currents (Fig. 4A). When GIRK1* was expressed alone, phosducin did not significantly reduce I_basal_ (Fig. 4D). We assume that, although Gβγ significantly contributes to I_basal_ in this channel^18^, the expected reduction in I_basal_ was obscured because of the relatively low I_basal_ observed in this experiment. In all other test groups, injection of His-phosducin into oocytes decreased GIRK1* current (Fig. 4B–D): by 74% for GIRK1* coexpressed with Gβγ, by 79% for GIRK1* coexpressed with YFP-Gγ, and by 63% for GIRK1* coexpressed with Gβ + YFP-Gγ (Fig. 4D). The inhibition of Gγ-YFP – induced GIRK1* activity suggests that the effect of Gγ depends on the presence of endogenous (ambient) Gβ.

**Figure 5:**
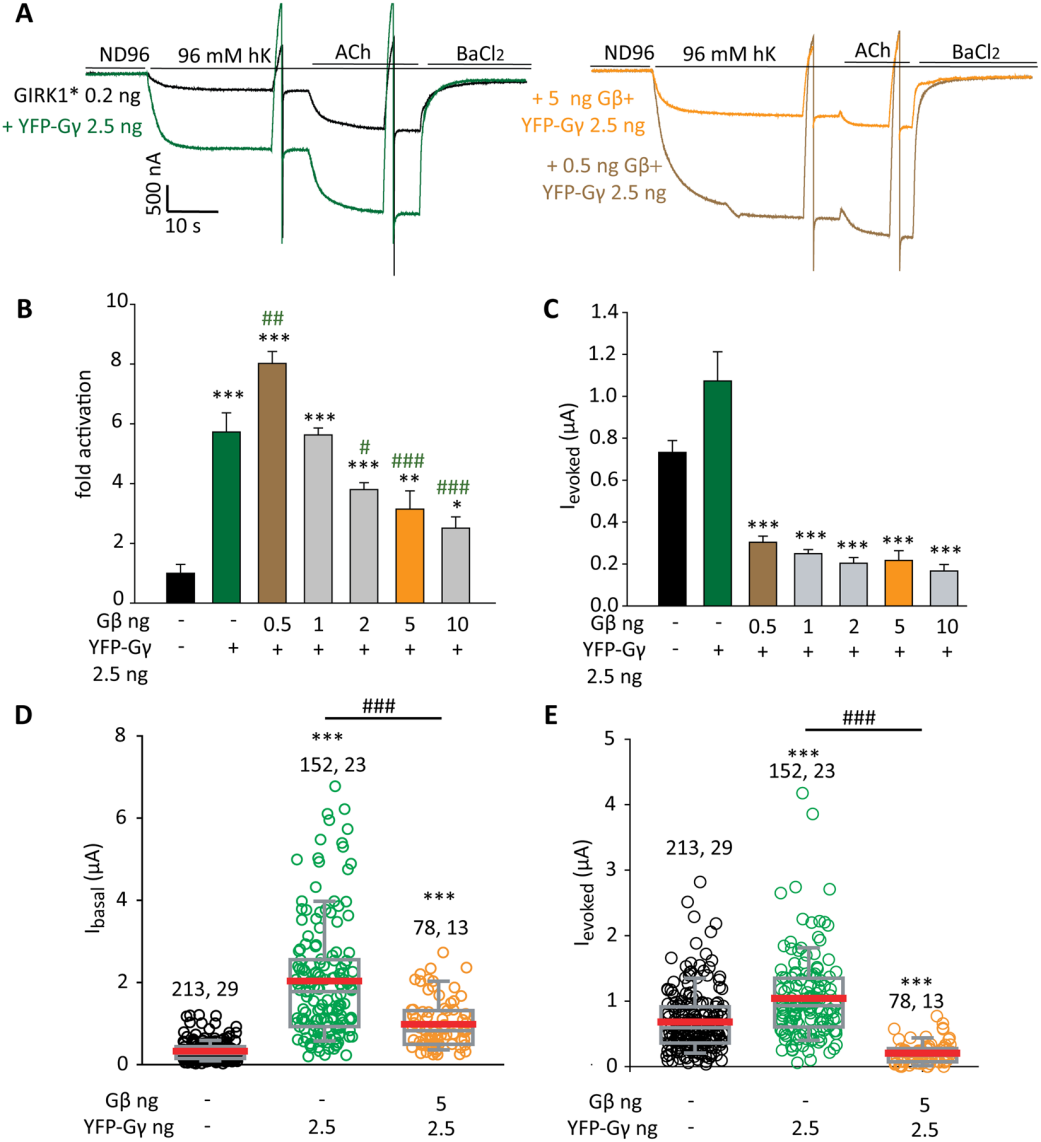
Complex stoichiometric relationships between Gγ and Gβ. (**A–C**) show one experiment in which RNA of Gβ was varied whilst RNA of YFP-Gγ was constant, 2.5 ng RNA/oocyte. m2R (1 ng RNA) was coexpressed in all cases. (**A**) Representative current records in oocytes expressing GIRK1* (0.2 ng RNA) alone or with YFP-Gγ, without or with Gβ (0.5 or 5 ng RNA). Note that the basal current was larger when YFP-Gγ was coexpressed with 0.5 ng Gβ than with 5 ng Gβ RNA. The sharp deflections in traces are currents elicited by voltage ramps used to obtain current-voltage curves, which are not shown. (**B**) Summary of fold activation of GIRK1* (0.2 ng RNA) by Gβγ or Gγ. n = 7 to 9 oocytes in each group, N = 1 experiment. Statistical analysis: asterisks * show difference from channel alone, pound signs # show difference from GIRK1 + YFP-Gγ (without Gβ). Compared to YFP-Gγ alone, coexpression of 0.5 ng Gβ significantly increased I_basal_ (p = 0.003) but coexpression of 5 ng Gβ reduced it (p < 0.001). One-way ANOVA (normal distribution) followed by Dunnett’s test. (**C**) Summary of I_evoked_. Expression of Gγ alone significantly elevated I_evoked_ (p < 0.001 by t-test). Comparison of groups expressing Gβ and Gγ vs. GIRK1* + YFP-Gγ (green bar) was done using one-way ANOVA followed by Dunnett’s test. ***p < 0.001. (**D**,**E**) show summary of the effects of YFP-Gγ vs. Gβ + YFP-Gγ on I_basal_ and I_evoked_ of GIRK1* (0.2 ng RNA) from all experiments described in this report. Numbers at the top are n, N (number of cells, number of experiments). Statistical analysis: one-way ANOVA followed by Dunn’s test. ***p < 0.001 relative to GIRK1*alone. T-test was done to compare between GIRK1* co-expressed with YFP-Gγ vs. GIRK1* with Gβ + YFP-Gγ; ^###^p < 0.001.

An additional way of using phosducin was coexpression of myristoylated phosducin (myr-phosducin) by the injection of its RNA into the oocytes (Fig. 4E). The myristoylation tag at the N-terminus of myr-phosducin targets it to the membranes, including the PM^65^. Expression of myr-phosducin significantly decreased I_basal_ of GIRK1* alone (Fig. 4H), as reported previously^18^. Myr-phosducin also inhibited ~90% of the GIRK1* current activated by coexpressed YFP-Gγ (Fig. 4G,H). Strikingly, when GIRK1* was activated by YFP-Gγ with coexpressed Gβ (5 ng RNA), expression of myr-phosducin increased the current (Fig. 4F,H), or had no effect (Supplementary Fig. 3B). The possible reason for this seemingly paradoxical effect became clear only later, after titration of Gβ concentrations (see Fig. 5 and the Discussion). We assumed that the expressed amount of phosducin is not sufficient to fully sequester all expressed Gβ and therefore does not inhibit GIRK1* activation. In support, when we injected a lower dose of Gβ (0.5 ng instead of 5 ng as in Fig. 4) together with YFP-Gγ, activation of GIRK1* was very strong but phosducin almost completely inhibited it (Supplementary Fig. S3B). In summary, inhibition of YFP-Gγ – induced activation by coexpressed or added (as purified protein) phosducin, supports the notion that Gγ – induced activation of GIRK1* requires ambient Gβ.

### Complex stoichiometric relationships between Gγ and Gβ

To better understand the mutual dependence of actions of Gβ and Gγ on GIRK1*, we titrated Gβ RNA in the presence of a fixed concentration of YFP-Gγ RNA (2.5 ng/oocyte). As before, YFP-Gγ increased both I_basal_ and I_evoked_ (Fig. 5A–C). Activation of GIRK1* by YFP-Gγ was strongly affected by coexpression of Gβ in a complex manner. A low dose of Gβ (0.5 ng) enhanced the effect of YFP-Gγ but higher doses of Gβ reversed this effect. Gβγ still activated GIRK1*, but with higher levels of expressed Gβ the activation was even lower than with YFP-Gγ alone (Fig. 5B). Strikingly, expression of even the lowest dose of Gβ, 0.5 ng RNA, suppressed I_evoked_ (Fig. 5C). Figure 5D,E summarize the effects of coexpression of YFP-Gγ alone and with 5 ng Gβ from all experiments, showing that the reduction of YFP-Gγ – induced activation of GIRK1* by a high dose of Gβ, and the suppression of I_evoked_, were highly reproducible and significant. Similarly, low doses of Gβ potentiated activation of GIRK1* induced by WT Gγ or YFP_A207K_-Gγ, and this effect was diminished when the dose of Gβ was increased (Supplementary Fig. S4). These results support the notion of mutual dependence of action of Gγ and Gβ. It appears that overexpression of either Gβ (Fig. 5) or Gγ (Fig. 1) is counterproductive for optimal channel activation, possibly through sequestration of one subunit by an excess of the other one (see Discussion).

### Gγ activates GIRK1/3 but not GIRK2 or GIRK1/2

**Figure 6:**
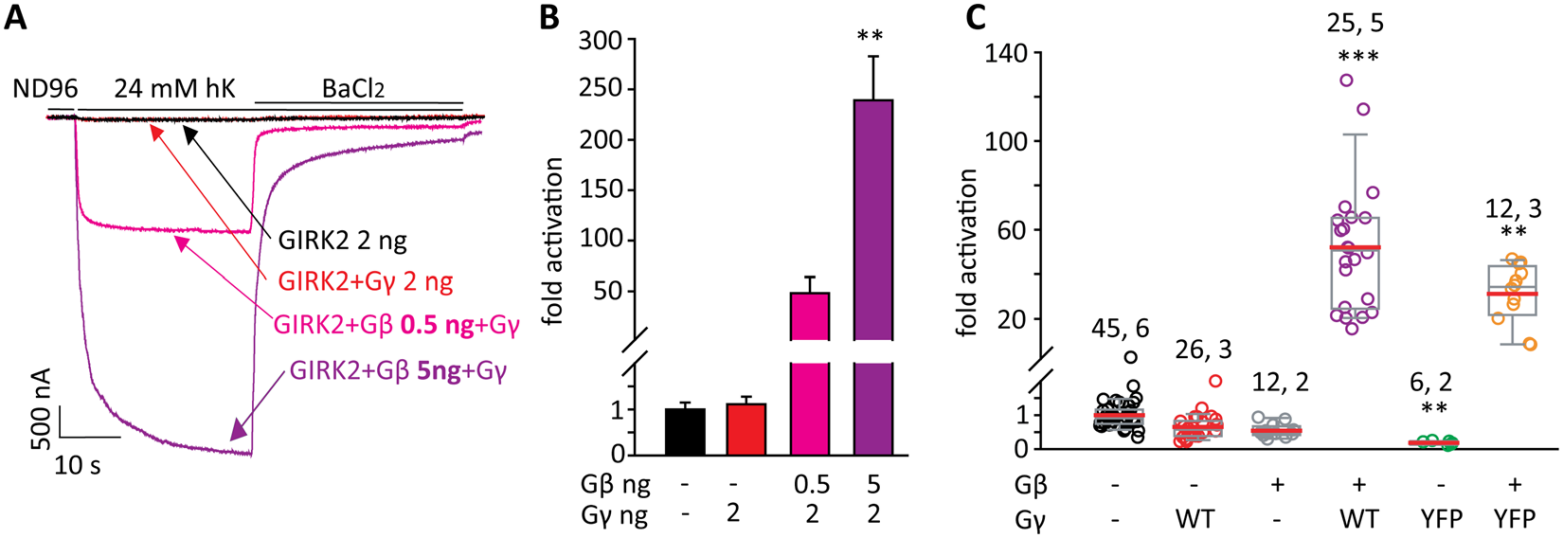
Gγ does not activate GIRK2. (**A**) Representative currents of GIRK2 (2 ng RNA) with two different concentrations of Gβ RNA and a fixed concentration of Gγ RNA, 2 ng/oocyte. (**B**) Summary the representative experiment shown in A, showing fold activation by Gγ or Gβγ in each group. One-way ANOVA followed by Dunn’s test. *, p < 0.05 relative to GIRK2 alone. n = 5–6 cells in each group; N = 1. (**C**) Summary of effects of Gβ, Gβγ, Gγ and YFP-Gγ from this series of experiments. RNA doses, in ng/oocyte, were: GIRK2, 2; Gγ, 1 or 2; Gβ, 5; YFP-Gγ, 2.5. Numbers above data sets are n, N (number of cells, number of experiments). Statistical analysis: one-way ANOVA followed by Dunn’s test. **p < 0.01, ***p < 0.001 relative to GIRK2 alone.

We explored whether Gγ affects neuronal GIRK channels of different subunit composition, starting with GIRK2. In contrast to GIRK1*, GIRK2 was not activated by Gγ and showed a canonical activation by Gβγ, with greater extent of activation with higher Gβ concentrations (Fig. 6A,B). YFP-Gγ also did not activate GIRK2 (Fig. 6C). Co-expression of Gβ with YFP-Gγ increased I_βγ_ as expected and to a similar extent as Gβ coexpressed with WT-Gγ (Fig. 6C). The small decrease in I_basal_ of GIRK2 by YFP-Gγ (Fig. 6C) may be due to changes in channel expression. These results indicate a specific role of GIRK1 subunit in mediating the effect of Gγ.

**Figure 7:**
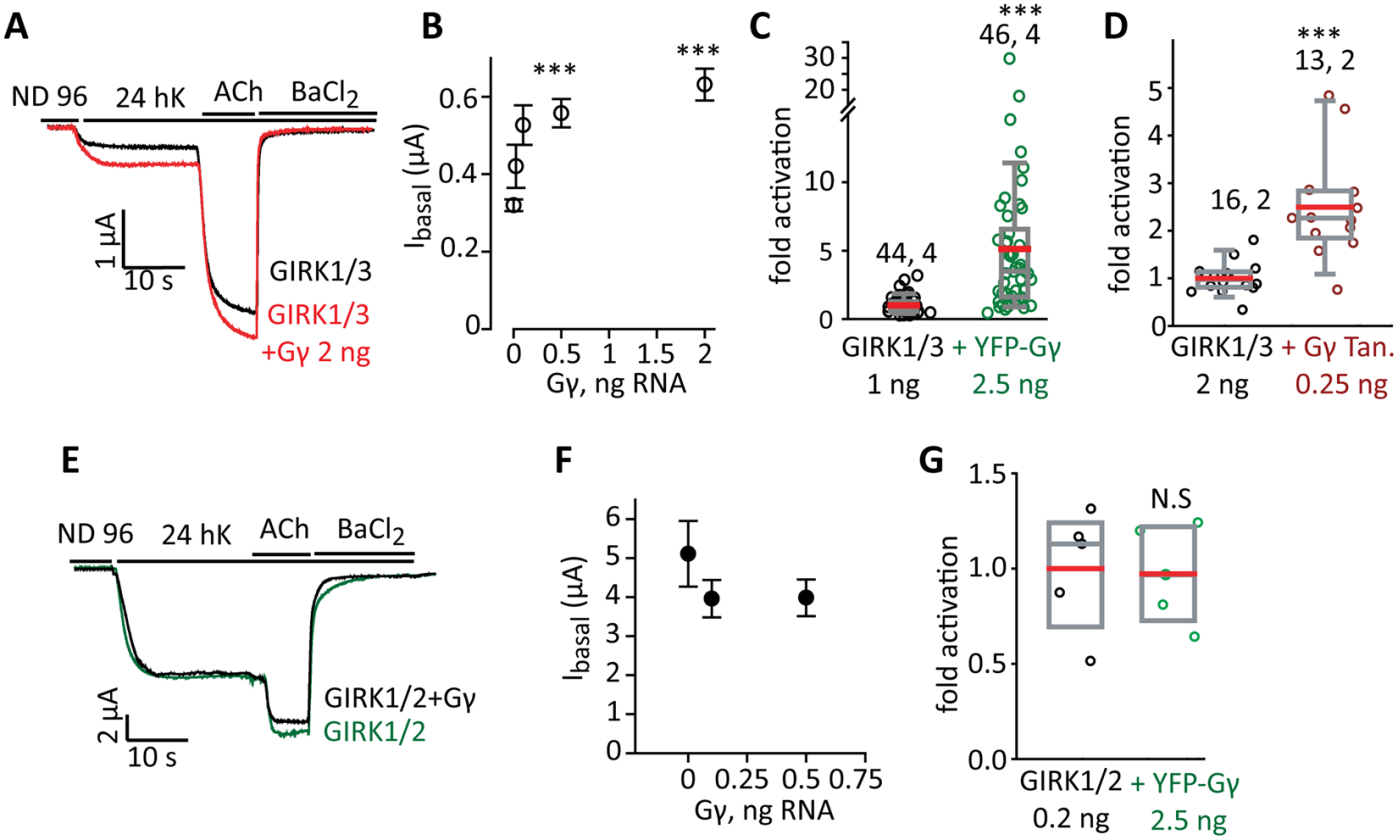
Gγ activates GIRK1/3 but not GIRK1/2. (**A,B**) Dose dependent activation of GIRK1/3 (1 ng RNA of each subunit) by Gγ (0.025-2 ng RNA). (**A**) Shows representative currents and (**B**) shows the full result of the experiment. n = 14 oocytes with each dose of Gγ. Statistics: one-way ANOVA followed by Dunnett’s test. ***p < 0.001 vs. GIRK1/3 alone. (**C**,**D**) Summary of effects of YFP-Gγ and Gγ tandem (Gγ Tan) on GIRK1/3. Numbers above data sets are n, N (number of cells, number of experiments). Statistics: t-test. (**E,F**) GIRK1/2 is not activated by Gγ. Measurements were done in the same experiment as in **A,B**; the effect of Gγ on GIRK1/3 served as positive control. GIRK1/2 (0.05 ng RNA of each subunit) was coexpressed without or with two RNA doses of Gγ, 0.1 or 0.5 ng. (**E**) Shows representative currents and (**F**) the full result of the experiment (5–9 cells in each group; N = 1). There was no significant difference between the groups. (**G**) YFP-Gγ does not activate GIRK1/2. There was no significant difference between the groups (n = 5, N = 1).

Next, we tested the effects of Gγ on GIRK1/2 and GIRK1/3, the most abundant neuronal GIRK channels. Fig. 7A,B shows a representative experiment in which GIRK1/3 was co-expressed with increasing doses of Gγ RNA. Gγ significantly increased I_basal_ up to a ~2 fold at 2 ng Gγ RNA (Fig. 7A,B). Interestingly, unlike in GIRK1*, here we did not observe the bell-shaped dose-response relationship, but we have not tested higher doses of Gγ. YFP-Gγ and Gγ tandem also significantly increased GIRK1/3 I_basal_, about 5- and 2.5 fold, respectively (Fig. 7C,D). I_evoked_ was not significantly affected by WT-Gγ (104 ± 9% of control, n = 12, N = 2, not shown) but it was significantly increased by YFP-Gγ (153 ± 11%, p < 0.001, n = 46, N = 6, not shown). In a separate experiment, we have monitored the effect of Gγ tandem on surface expression of GIRK1/3 in giant excised PM patches (Supplementary Fig. S5). Titrated expression of RNA of the Gγ tandem indicated that at concentration that produced maximal activation of GIRK1/3 (0.25 ng RNA, Supplementary Fig. S5C), the Gγ tandem did not affect the level of GIRK1/3 in the PM. These experiments suggest that coexpression of Gγ increases GIRK1/3 currents without affecting the channel’s surface levels.

Unlike GIRK1* or GIRK1/3, the basal activity of GIRK1/2 was not affected by coexpression of Gγ or YFP-Gγ (Fig. 7E–G). I_evoked_ was also unaffected (not shown). Note that I_basal_ of GIRK1/2 was > 4 µA (Fig. 7E,F) with a low dose of RNA, 0.05 ng RNA of each subunit. I_basal_ of GIRK2 was 0.36 ± 0.05 µA (n = 45) with a 40-fold higher dose of RNA, 2 ng/oocyte (for review on I_basal_ differences in GIRK channels, see ref.^29^). Therefore, >90% of I_basal_ in this case originated from GIRK1/2 heterotetramers rather than from any incidentally present GIRK2 homotetramers. We conclude that GIRK2 is not activated by Gγ, and it also appears to prevent Gγ-induced activation of GIRK1 in a GIRK1/2 heterotetrameric context.

### Distal C-terminus of GIRK1 (dCT) is important for Gγ-induced activation

We hypothesized that a structural difference between GIRK1* and GIRK2 may explain the divergent effects of Gγ on GIRK1* and GIRK2 in the homomeric context. One candidate structural element is the unique distal C-terminus of GIRK1, G1-dCT, which contributes to Gβγ anchoring and channel gating^29^. Both GIRK2 and GIRK1*_Δ121_ (which is a deletion mutant of GIRK1* without the dCT) lack this element and cannot recruit Gβγ. Accordingly, they have low basal activity and stronger relative activation by Gβγ compared to GIRK1* and GIRK1/2, suggesting that the Gβγ activation site in channel’s core is intact^18,34^.

**Figure 8:**
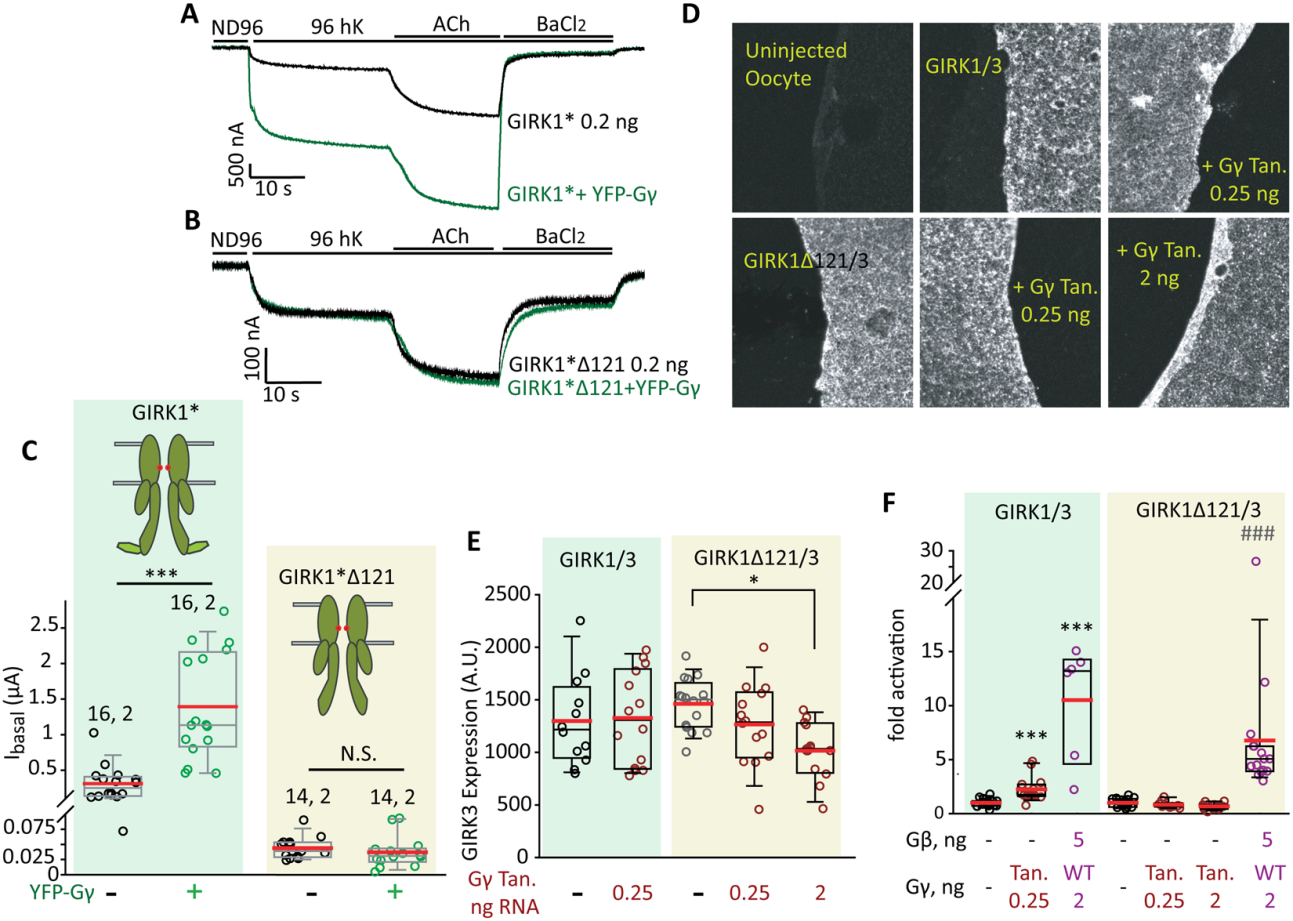
Distal C-terminus of GIRK1 is important for Gγ activation of GIRK1* and GIRK1/3. (**A–C**) Deletion of dCT abolishes the activating effect of YFP-Gγ (2.5 ng RNA) on GIRK1*. (**A**,**B**) show representative traces of GIRK1* (**A**) and GIRK1*_Δ121_ (**B**) expressed alone or with YFP-Gγ. m2R was expressed in all cases. (**C**) Summary of the experiment. Numbers above data sets are n, N. Statistics: t-test for each pair with and without YFP-Gγ. ***p < 0.001, N.S., not statistically significant. (**D**–**F**) Expression of Gγ tandem (Gγ Tan) does not affect the expression of GIRK1/3 in the PM (**D,E**) and activates GIRK1/3 but not GIRK1_Δ121_/3 (GIRK1_Δ121_/3; **F**). Oocytes were stained with an antibody against GIRK3. Representative images of giant excised PM patches are shown in (**D**) and summary of measurements in (**E**) Gγ tandem did not affect the expression of GIRK1/3, but reduced the expression of GIRK1_Δ121_/3 when expressed at a high dose (p < 0.05 for 2 ng Gγ tandem). A.U., arbitrary units. n = 12–16 membranes in each group, N = 1. (**F**) Summary of the effects of Gγ tandem and of Gβγ on I_basal_. n = 10–15 cells in each group, N = 2. Gγ tandem and Gβγ significantly increased I_basal_ of GIRK1/3 (***p ≤ 0.001 vs. GIRK1/3 alone). GIRK1_Δ121_/3 was not affected by Gγ tandem but was activated by Gβγ (###p < 0.001 vs. GIRK1_Δ121_/3 alone).

To assess the possible role of G1-dCT, we coexpressed YFP-Gγ with GIRK1*_Δ121_. Figure 8A,B shows an exemplary experiment, Fig. 8C summarizes two experiments of this kind. YFP-Gγ had no effect on GIRK1*_Δ121_ expressed at low density (0.2 ng RNA), whereas YFP-Gγ increased GIRK1* I_basal_ as expected (Fig. 8A–C). When GIRK1*_Δ121_ was expressed at a higher level, 2 ng RNA, WT Gγ did not enhance I_basal_, but YFP-Gγ appeared to produce a residual 2-fold activation of GIRK1*_Δ121_ that did not reach statistical significance in one-way ANOVA test (Supplementary Fig. S6A). Notably, both WT Gγ and YFP-Gγ significantly reduced I_evoked_ of GIRK1_Δ121_*, further underscoring the importance of G1-dCT for Gγ regulation of GIRK1* (Supplementary Fig. S6B). We also tested GIRK1_Δ121_ as a heterotetramer with GIRK3, GIRK1_Δ121_/3. This channel expressed well and its level in the PM was similar to that of GIRK1/3 containing the full-length GIRK1 subunit (Fig. 8D,E). Coexpression of Gγ tandem did not change the PM level of GIRK1/3; the PM level of GIRK1_Δ121_/GIRK3 was reduced by ~20% by the higher dose of Gγ tandem used, 2 ng RNA/oocyte (Fig. 8D,E). Unlike GIRK1/3, GIRK1_Δ121_/3 was not activated by coexpression of Gγ tandem (Fig. 8F). Gβγ strongly activated both GIRK1/3 and GIRK1_Δ121_/3 (Fig. 8F). Thus, removal of the G1-dCT significantly reduces the activating effect of Gγ on GIRK1* and GIRK1/3, suggesting a role for G1-dCT in this action of Gγ.

**Figure 9:**
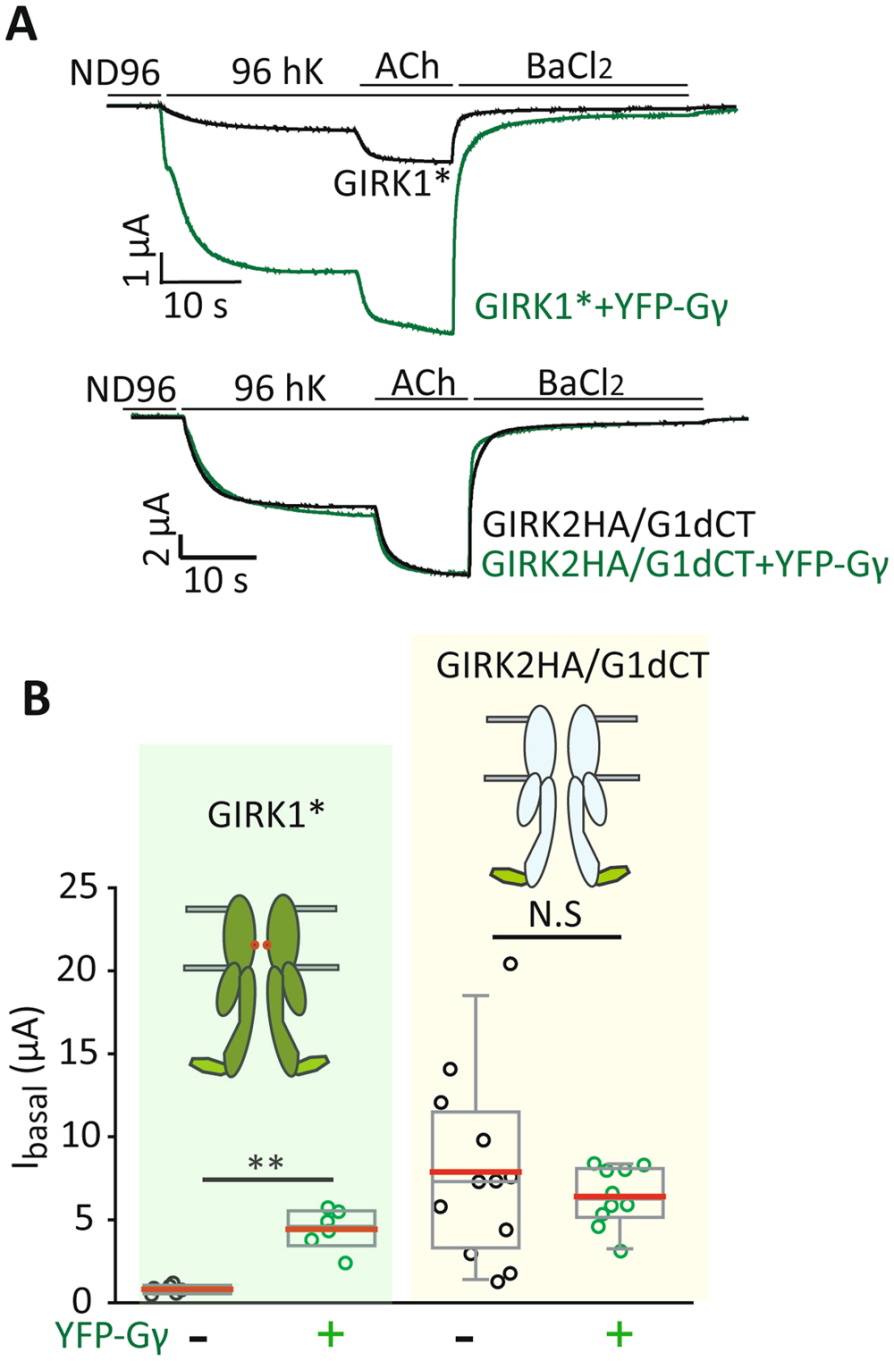
Addition of G1-dCT to GIRK2 does not confer Gγ sensitivity. (**A**) Representative traces of GIRK1* (top) or GIRK2HA/G1-dCT (bottom), expressed alone (0.2 ng RNA) or with YFP-Gγ (2.5 ng RNA). (**B**) Summary of the effect of YFP-Gγ on I_basal_. YFP-Gγ activates GIRK1* but not GIRK2HA/G1-dCT. GIRK1*: n = 6, chimera alone: n = 12, chimera with YFP-Gγ: n = 10, N = 1. **, p < 0.01, N.S. - not statistically significant (t-test for each construct with and without YFP-Gγ).

We next wanted to test whether addition of G1-dCT to GIRK2 will render the channel sensitive to Gγ. For this purpose, we used the chimera GIRK2HA/GIRK1dCT, which contains the G1-dCT (a.a. 371–501) fused to dCT-less GIRK2 (a.a. 1–381), as well as an extracellular HA tag (see cartoon in Fig. 9B). We have previously shown that C-terminal fusion of G1-dCT confers upon GIRK2HA the ability to recruit Gβγ to the PM; accordingly, this chimera has a much greater I_basal_ than GIRK2HA^18,34^. However, YFP-Gγ did not activate the GIRK2HA/G1-dCT channel. In the same experiment, YFP-Gγ increased GIRK1* I_basal_ as expected (Fig. 9A,B). This result indicates that although G1-dCT is necessary for Gγ effect, it is not sufficient and possibly requires additional structural elements of the channel.

## Discussion

In performing its cellular functions, Gβγ acts as an obligatory, stable dimer, dissociated only by denaturation^43,70^. Gβ and Gγ are synthesized separately^59,71^; dimerization greatly increases the stability of each subunit^72–74^. Folding of Gβ requires the chaperone CCT complex, from which Gβ is released by the co-chaperone PhLP1^59,71^. Mature Gβγ dimer is formed only after the binding of Gγ^75^. Lipid modification (prenylation) of Gγ is not required for association with Gβ, but is crucial for PM targeting of Gβγ and for activation of GIRKs^45,46^. In accord with these cellular mechanisms, we found that heterologous expression of Gβ alone did not activate GIRK1* or GIRK2 channels (Figs 2, 6, S6).

Unlike Gβ, Gγ can fold separately and is produced in cells in the absence of Gβ^71,76^, especially when fused to GFP, though still less than with Gβ^60^. We found that overexpression of Gγ or Gγ tagged with CFP or YFP enhances the activity of homomeric GIRK1* and heterotetrameric GIRK1/3 channels. Interestingly, the strongest activation was achieved by YFP-Gγ without the A207K mutation, where YFP is prone to dimer formation^63^. Gγ tandem (concatemer) consisting of two fused Gγ subunits activated GIRK1* and GIRK1/3 similarly to YFP-Gγ (Figs 3, 7, 8), excluding a role for the xFP moiety in channel activation. No natural Gγ dimer formation has been reported; but we hypothesize that artificial dimerization can protect against degradation and increase Gγ’s stability. Alternatively, dimerization could be related to some steric aspect of the mechanism of action of Gγ.

To address the action of Gγ, we first considered the possibility that expression of Gγ facilitates synthesis or trafficking to PM of GIRK1, which could account for the observed increase in I_basal_ and I_evoked_. However, direct immunocytochemical measurements in giant excised PM patches consistently showed no change in the amount of GIRK1* and GIRK1/3 channel protein in the PM (Figs 3, 8, S5).

We then hypothesized that the expressed Gγ may “recruit” Gβ to the PM. Absence of GIRK activation by expression of Gβ alone suggested that there was no free ambient (endogenous) Gγ to form functional dimers with the expressed Gβ. It was plausible, however, that expression of exogenous Gγ could enhance the release of ambient (endogenous) Gβ trapped in the CCT^75^, and in this way elevate the total Gβγ in the PM. However, several lines of evidence argue against the Gβ recruitment hypothesis. First, we directly measured the PM levels of endogenous Gβ using Western blots of manually separated PMs. Expression of Gγ at doses that produced robust activation of GIRK1* did not significantly change PM levels of Gβ (Fig. 1). Second, Gγ and its derivatives did not decrease or (for low GIRK1* expression levels) increased I_evoked_ of GIRK1*, whereas expression of Gβγ always decreased it (Figs 2, 5, S2). This is incompatible with recruitment: even a small addition of Gβ (if it were recruited by Gγ) on top of Gγ would reduce I_evoked_ in GIRK1* (see Fig. 5). Third, expression of Gγ or YFP-Gγ did not activate either GIRK2, which is highly sensitive to expressed Gβγ^18^ (Fig. 6), or GIRK1*_Δ121_ that retains the GIRK1 Gβγ-activation site and is strongly activated by Gβγ^34^, or GIRK1_Δ121_/3 (Fig. 8). The inability of Gγ to activate these channels strongly argues against the Gβ-recruitment hypothesis. All evidence considered, we conclude that changes in PM levels of either GIRK or Gβγ cannot explain the activating effect of Gγ. We therefore propose that Gγ regulates the gating of GIRK1* and GIRK1/3.

How can Gγ regulate GIRK gating? A key insight into the mechanism of Gγ action comes from the finding that Gγ action is blocked by phosducin (Fig. 4). Phosducin interacts with Gβ but not with Gγ^68,69^, implicating Gβ in the effect of Gγ. Another indication of the involvement of Gβ is the enhancement of Gγ effect by coexpression of low doses of Gβ (Figs 5, S4). Therefore, we suggest that the presence of ambient Gβ is essential for the activation of GIRK1* and GIRK1/3 by Gγ. Since the presence of free Gβ in cells is unlikely, we propose that the effect of Gγ requires the presence of ambient Gβγ, which is dynamically associated with the GIRK1-containing channels^29^.

Another key insight came from the lack of GIRK2 activation by Gγ (Fig. 6). It implied the possible involvement of G1-dCT, which is unique to GIRK1, in Gγ regulation of GIRK1* and GIRK1/3. In support, deletion of G1-dCT greatly reduced the Gγ-induced activation of GIRK1* and GIRK1/3 (Figs 7, 8, S6) and completely abolished the Gγ-induced increase in I_evoked_, causing a decrease instead (Fig. S6). To further address the function of G1-dCT, we used the chimera G2HA/G1-dCT in which the short dCT of GIRK2 (~34 amino acids) is replaced by G1-dCT. Addition of G1-dCT endows this chimera with an enhanced Gβγ binding compared to GIRK2; it also recruits Gβγ to the PM^34^. However, the G2HA/G1-dCT channel was not affected by Gγ (Fig. 9), suggesting that G1-dCT alone is not sufficient to confer Gγ activation to GIRK2. Core elements in GIRK1 may also be involved; indeed, cross-talk between gating effects of G1-dCT and core of GIRK1 has been proposed^31^. Furthermore, since the G2HA/G1-dCT channel recruits Gβγ, we posit that Gγ activation is not related to recruitment of Gβγ. We hypothesize that it may be related to the second function of the G1-dCT, which is an inhibitory one, as explained below.

**Figure 10:**
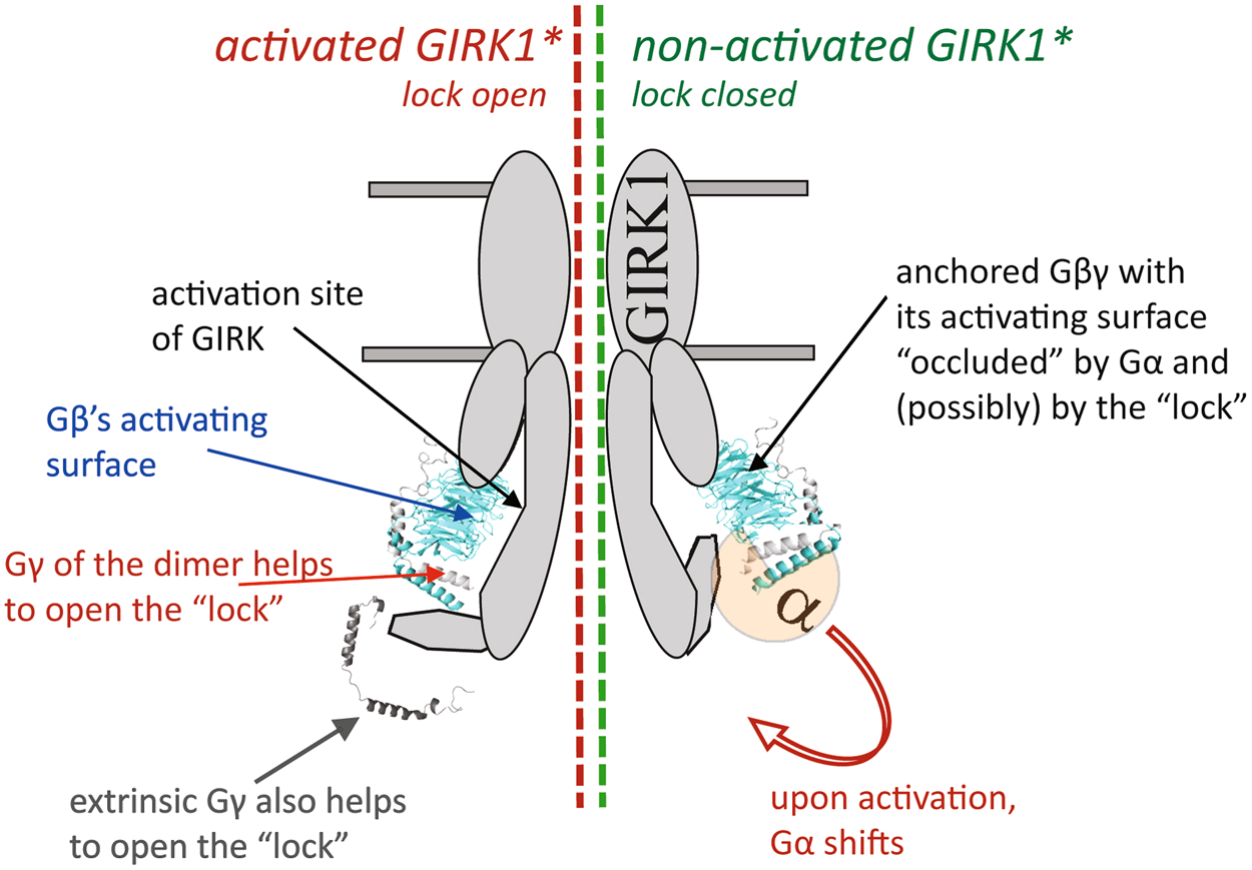
A hypothetical scheme of regulation of a GIRK channel containing the GIRK1 subunit by Gγ. In GIRK1-containing channels, Gβγ or the Gαβγ heterotrimer (shown in the figure) may be anchored to GIRK. In the resting state, the interaction surface of Gβ is occluded by Gα and cannot contact the activation site of GIRK. Lock element (encompassing the G1-dCT and other unknown parts of the channel) is closed, reducing channel activity. Upon activation by agonist, the GPCR (not shown) activates the G protein causing dissociation of Gα from Gβγ, exposing the GIRK-interacting surface of Gβ. Gβγ may now bind to the activation site. We propose that, at the same time, Gγ interacts with a channel’s element and helps to release the inhibitory effect of the “lock”. Exogenous Gγ may mimic this action without activating the channel by itself, but only if Gβγ is present.

In light of these considerations, we propose a model (Fig. 10) in which Gγ aids Gβ to drive the opening of GIRK1* and GIRK1/3 by acting on a gating element within the channel, rather than by participating in binding to the activation site. We envision that this function is normally carried out by Gγ from within the Gβγ dimer, e.g. when Gβγ is released from the Gαβγ heterotrimer following GPCR-catalyzed GDP-GTP exchange (Fig. 10, “activated GIRK1*). We propose that the coexpressed, properly prenylated Gγ can reach the vicinity of the channel and act on the same gating element, further helping Gβγ to shift the closed-open equilibrium in favor of the open state. Since GIRK1/2 is not regulated by Gγ, we propose that GIRK2 either counteracts or occludes the action of Gγ; this will be discussed separately.

The nature of the gating element affected by Gγ is currently unclear. As a working hypothesis, we put forward the involvement of the hypothetical “lock” encompassed, in part, by the G1-dCT. A peptide corresponding to the last 20 amino acids of GIRK1 reduced the open probability (P_o_) of Gβγ-activated GIRK1/5 and GIRK1/4 channels by a non-competitive mechanism, suggesting a gating effect rather than competition for Gβγ binding^36^. Accordingly, the “lock”-deficient GIRK1*_Δ121_ has a higher maximal Gβγ-induced P_o_ that GIRK1*^34^. Thus, one of the possible scenarios of Gγ action could be the removal of the inhibitory effect of the “lock”, which would increase both I_basal_ and I_evoked_ by increasing the P_o_ of Gβγ-activated channels. We emphasize that other mechanisms in which Gγ allosterically regulates (enhances) the P_o_ will result in the same action. The proposed mechanism helps to explain, and is supported by, the findings obtained used the “expression pharmacology” approach^62,77^ (titration of protein expression in oocytes by injecting a range of RNA doses).

Finding #1: Gγ increases I_basal_ and I_evoked_ of GIRK1* and GIRK1/3, but expression of free Gβγ suppresses I_evoked_ (Figs 1–5). Explanation: Gγ acts by enhancing Gβγ-induced activation, irrespective of the source of Gβγ (a pre-associated “basal” Gβγ or Gβγ released from Gαβγ through the activation of GPCR). Hence the increase both in I_basal_ and I_evoked_. In contrast, coexpressed Gβγ suppresses I_evoked_ because it already maximally activates GIRK channels^21^, and also sequesters Gα away from the GIRK-G protein complex, reducing activation by the GPCR^18^.

Finding #2: Expression of small amounts of Gβ enhances the Gγ-induced activation better than higher amounts of Gβ (Figs 5, S2). Explanation: GIRK1-containing channels are associated with excess of Gβγ over Gα^29^, possibly already with 3–3.5 Gβγ molecules per channel^62^. A low dose of added Gβγ may suffice to reach the maximal stoichiometry of 4 Gβγ per one GIRK1* channel. Under these conditions, Gγ further increases P_o_ by further shifting closed-open equilibrium in favor of the open state. Expression of excess Gβ and formation of more Gβγ dimers cannot cause further activation. However, it might decrease (sequester) free Gγ and reduce its concentration, counteracting the enhancing action of Gγ.

Finding #3: Expression of high doses of Gγ produces a smaller activation of GIRK1* than an optimal, lower dose (Figs 1, 3). Explanation: similarly to the excess of Gβ, excess Gγ may be sequestering Gβ “away from the channel”. However, more complex mechanisms cannot be excluded.

Finding #4: In the presence of coexpressed high dose of Gβγ, expression of phosducin further increased I_basal_ (Fig. 4H). Rather counterintuitive, this finding is consistent with the proposed mechanistic framework. With 5 ng phosducin RNA, sequestration of Gβγ is probably incomplete^56,65^ and leaves a small amount of “extra” expressed Gβγ. The latter, together with added coexpressed Gγ, activates the channel to a high level, as explained above. In line with this, with low dose of Gβ RNA (0.5 ng) phosducin effectively counteracted the action of Gγ, supporting the role of Gβ and underscoring the importance of stoichiometric considerations. Interestingly, the injected purified phosducin decreased channel activation in all conditions, suggesting a more complete Gβγ sequestration. Other possibilities, such as a long-term effect of coexpressed myr-phosducin on cellular levels of Gβγ or interactions within the signaling complex, cannot be ruled out.

In addition to mutual sequestration of Gβ, Gγ and phosducin, mechanisms that could contribute to the reduction in Gγ activation at high Gγ or Gβ levels include the formation of inactive Gβγ oligomers or Gγ aggregates^74^, or variation in stoichiometry of Gα that may alter channel’s activity. In summary, we have found that stoichiometric relationships between expressed proteins crucially determine the observed regulations of GIRKs by Gβ and Gγ. Mutual sequestration or formation of protein oligomers of inadequate stoichiometry probably explain the complex, bell-shaped dose-response relationships of Gγ and Gβ effects (Figs 1, 3, 5, S2). The new insights obtained here underscore the power of the “expression pharmacology” approach for studies of complex regulatory mechanisms in heterologous expression systems.

Importantly, GIRKs of different subunit composition showed diverse regulation by Gγ. Only GIRK1/3 was regulated similarly to GIRK1*; GIRK2 and GIRK1/2 were not affected by Gγ. These findings carry a potential physiological relevance because of the specific and diverse subunit composition of GIRKs in the brain. Furthermore, they may provide new insights as to the mechanism of Gγ action and, generally, of the regulation of GIRKs by G protein subunits. If, as we have proposed, Gγ allosterically regulates GIRK by acting on a gating element (such as the “lock” present in GIRK1), then GIRK2 may contain a structural element that exerts the same effect, whereas GIRK3 does not. In this way GIRK2 may occlude the effect of Gγ. This would explain why GIRK1/3 is regulated by coexpression of Gγ whereas GIRK1/2 is not. Another possibility is that GIRK2 counteracts the effect of Gγ by acting on a different structural element within the GIRK1/2 heterotetramer.

Absence of activation of GIRK1/2 by coexpressed Gγ seems to be at odds with the previous finding that Gγ is essential for Gβ to activate GIRK1/2^54^. However, a more detailed examination of our and Kawano’s data reveals that there is no controversy. Kawano *et al*.^54^ showed that mutated Gβ that does not bind Gγ can still associate (at least it is co-immunoprecipitated) with GIRK subunits, but it cannot activate GIRK1/2. These results are supported by our data where Gβ alone cannot activate any of the GIRK channels tested, and corroborate the notion that, without prenylation of Gγ, Gβγ cannot reach the PM and cannot activate any GIRK channel^45,46^. In comparison, our approach with expression of Gγ reveals unknown mechanistic aspects of Gγ action with physiologically relevant, functional Gβ and Gγ proteins. Our results show that Gγ not only is essential for GIRK activation by the Gβγ dimer, but also actively supports the Gβγ-induced transition to open state.

### Conclusions

We demonstrate that the Gγ subunit contributes to Gβγ-induced activation of GIRK channels in a GIRK subunit-specific manner. Expression of Gγ alone activated homotetrameric GIRK1* and heterotetrameric GIRK1/3 channels, but not GIRK2 or GIRK1/2. In GIRK1* and GIRK1/3, Gγ increases both I_basal_ and I_evoked_, without affecting surface expression of the channels. Our results suggest that, besides its known role in targeting Gβγ to the plasma membrane, Gγ regulates the gating of GIRKs, in concert with Gβ. The unique distal C-terminus of GIRK1, G1-dCT, is important but not sufficient for Gγ action. As a working hypothesis, we propose that Gγ regulates GIRK1* and GIRK1/3 channels by relaxing the inhibitory effect of the “lock” which is encompassed, in part, by the G1-dCT. We further hypothesize that, within the GIRK1/2 heterotetramer, GIRK2 acts to occlude the effect of Gγ, either by operating through the same mechanism as Gγ, or by triggering an opposing gating effect.

## Methods

### Ethical approval and animals

Experiments were approved by Tel Aviv University Institutional Animal Care and Use Committee (permits M-08-081 and M-13-002). All experiments were performed in accordance with relevant guidelines and regulations. *Xenopus laevis* female frogs were maintained and operated as described^78^. Frogs were kept in dechlorinated water tanks at 20 ± 2 °C on 10 h light/14 h dark cycle, anesthetized in a 0.17% solution of procaine methanesulphonate (MS222), and portions of ovary were removed through a small incision in the abdomen. The incision was sutured, and the animal was held in a separate tank until it had fully recovered from the anesthesia and then returned to post-operational animals’ tank. The animals did not show any signs of post-operational distress and were allowed to recover for at least 3 months until the next surgery. Following the final collection of oocytes, after 4 surgeries at most, anesthetized frogs were killed by decapitation and double pithing.

### DNA constructs, RNA and purified phosducin

cDNA constructs of YFP- or CFP- labeled and unlabeled GIRK subunits, Gβ_1_, Gγ_2_, m2R and myristoylated phosducin (myr-Phosducin) constructs were cloned into pGEM-HE, pGEM-HJ or pBS-MXT vectors, which are high expression oocyte vectors containing 5′ and 3′ untranslated sequences of Xenopus β-globin^79^, as described^65,80^. Constructs are described in Table 1. All PCR products were fully sequenced. Fluorescent xFP proteins (CFP_A207K_ and YFP_A207K_) usually contained the A207K mutation that prevents their dimerization^63^; however, when indicated, YFP was also used without the A207K mutation. Point mutations were introduced using PCR with the Pwo Master polymerase (Roche) according to manufacturer’s instructions, with primers containing the desired mutation. Afterwards, DpnI (New England Biolabs, R0176) was added to the reaction in order to degrade the template. The cDNA constructs were fully sequenced.

**Table 1:** List of cDNA constructs.

Construct	Species	Accession #	Comments
GIRK1*	Human	NM_002239	a pore mutation in GIRK1, F137S, allows its expression as a homotetramer
GIRK1	Rat	NM_031610.3	
GIRK1*_Δ121_	Rat	NM_031610.3	Lacks the last 121 a.a., aka the G1-dCT
GIRK2	Mouse	NM_002240.4	
GIRK3	Rat	NM_053834.1	
GIRK2-dCTG1	Mouse (GIRK2), Rat (GIRK1)	NM_031610.3 (GIRK1) NM_002240.4 (GIRK2)	Chimera consist of GIRK2 (a.a. 1-381) and GIRK1 (a.a. 371–501).
Gβ_1_	Bovine	NM_175777.3	
YFP-Gγ_2_	Bovine (Gγ)	NM_174072.4	YFP without the A207K mutation
YFP_A207K_-Gγ_2_	Bovine (Gγ)	NM_174072.4	YFP with the A207K mutation.
CFP_A207K_-Gγ_2_	Bovine (Gγ)	NM_174072.4	CFP with the A207K mutation.
Gγ_2_ tandem	Bovine	NM_174072.4	Concatemer of two Gγ_2_ subunit with the linker Ser-Arg (encoded by XbaI sequence, TCTAGA). Gγ_2_ tandem was constructed using PCR followed by ligation.
Gγ_2_	Bovine	NM_174072.4	
m2R	Human	NM_001006630.1	
myr-phosducin	Bovine	NM_001166527.1	Phosducin with myristoylation tag
His-phosducin	Bovine	NM_001166527.1	Used for protein production

RNA was transcribed *in vitro* essentially as described^78^ but precipitated overnight at −20 °C with 4 M LiCl instead of the standard ethanol/salt precipitation. RNA was divided into 1–2 μl aliquots and stored at −80 °C. The amounts of RNA injected per oocyte were varied according to the experimental design and are indicated in the results or in figure legends.

To prevent the formation of GIRK1*/GIRK5 heterotetramers, we injected the antisense oligonucleotide 5′T*A*AAT*CCC*TTG*CCA*TGA*T*G*G*T-3′ (*denotes phosphorothioate bond) targeted against the oocyte’s endogenous GIRK5 subunit of *Xenopus* GIRK5^81^ when studying GIRK1*, GIRK1/3 or GIRK chimeras.

For His-phosducin protein production, the coding sequence of bovine phosducin cDNA (see Table 1) was subcloned into pETMII vector which adds an N-terminal His-tag. Protein was amplified in BL-21 *E. coli*. Protein purification was done with Ni-NTA column using the following buffer: 50 mM KH_2_PO_4_, 20 mM Tris-HCl, 100 mM NaCl, 5 mM β-mercaptoethanol, 250 mM Imidazole. The size of His-phosducin is ~29 kDa and its initial concentration was 13.5 mg/ml, or 465 µM. The injection volume per oocyte was 50 nl, therefore the final concentration of His-phosducin was ~23 µM in each oocyte (assuming oocyte volume of ~1 µl).

### Electrophysiology

Oocyte defolliculation, incubation and RNA injection were performed as described previously^78^. Oocytes were defolliculated with collagenase (Type 1 A, Sigma) in Ca-free ND96 solution (see below) and injected with 50 nl of RNA, and incubated for 2–4 days in NDE solution (ND96 solution supplemented with 2.5 mM pyruvate and 50 µg/ml gentamicin) at 20 °C prior to testing. The standard ND96 solution contained (in mM): 96 NaCl, 2 KCl, 1 MgCl_2_, 1 CaCl_2_, 5 HEPES, and was titrated with NaOH to pH of 7.6–7.8. CaCl_2_ was omitted in Ca-free ND96.

Whole-cell GIRK currents in oocytes were measured using two-electrode voltage clamp (TEVC) with Geneclamp 500 (Molecular Devices, Sunnyvale, CA, USA), using agarose cushion electrodes^82^ filled with 3 M KCl, with resistances of 0.1–0.5 MΩ. GIRK currents were measured at a holding potential of −80 mV in either the standard low-[K^+^] ND96 solution, or in high [K^+^] solution (HK). We used HK with either 24 mM [K]_out_ (in mM: 24 KCl, 72 NaCl, 1 CaCl_2_, 1 MgCl_2_ and 5 HEPES) or 96 mM [K]_out_ (in mM: 96 KCl, 2 NaCl, 1 CaCl_2_, 1 MgCl_2_ and 5 HEPES). pH of all solutions was 7.4–7.6. Currents were measured as explained in Fig. 1A. Net basal GIRK currents were calculated by subtracting the residual Ba^2+^-insensitive current recorded in each cell at the end of the recording protocol, or, on rare occasions, by subtracting average current recorded in naïve oocytes of the same experiment. Data acquisition and analysis were performed using the pCLAMP 9 or pCLAMP 10 software (Molecular Devices, Sunnyvale, CA, USA).

### Measurement of Gβγ in plasma membrane (PM) by Western blotting

Plasma membranes were separated from the rest of the oocyte (“cytosol”) as described^61,83^. In brief, PM together with the vitelline membranes (extracellular collagen-like matrix) was removed manually with fine forceps after a 5–15 min incubation of the oocyte in a low osmolarity solution (5 mM NaCl, 5 mM HEPES, and protease inhibitors (Roche Complete Protease Inhibitors Cocktail, 1 tablet/50 ml), pH = 7.5). The remainder of the cell (“cytosol”) was processed separately, after removing of nuclei by centrifugation for 10 min at 700 × g at 4 °C. PMs (18–25 per lane) were solubilized in 35 μl running buffer (2% SDS, 10% glycerol, 5% β-mercaptoethanol, 0.05% Bromophenol Blue, 62.5 mM Tris-HCl, pH 6.8). Samples were electrophoresed on 12% polyacrylamide-SDS gel and transferred to nitrocellulose membranes for Western blotting with previously characterized^62^ antibody against Gβ at 1:500 or 1:1000 dilution (Santa Cruz Biotechnology, SC-378). Goat Anti-Rabbit IgG Antibody, (H + L) HRP conjugate secondary antibody at 1:40,000 dilution was applied (Merck Millipore, AP307P). The signals were visualized using the SuperSignal kit (Thermo, 15168) and images were obtained with the fluorescent imager Fusion FX7 (Vilber Lourmat, Germany) and quantitated using the ImageJ software (National Institutes of Health, USA). Oocytes of stage 6^84^) were used. These large cells have a rather constant size (~1 mm in diameter); the total amount of protein in the PM is considered uniform, and in Western blots of oocyte’s protein number of loaded oocytes rather than µg protein is routinely reported, and no normalization of measured amount of Gβ to a housekeeping protein is considered necessary (e.g. refs^61,62,83,85^). The same amount of cells was used for each lane on the gel (Fig. 1D, Supplementary Fig. S1).

### Giant excised PM patches

Giant excised PM patches were prepared, stained with antibodies and imaged as described^86^. Oocytes were mechanically devitellinized using fine forceps in a hypertonic solution (in mM: NaCl 6, KCl 150, MgCl_2_ 4, HEPES 10, pH 7.6). The devitellinized oocytes were transferred onto a Thermanox™ coverslip (Nunc, Roskilde, Denmark) immersed in a Ca^2+^-free ND96 solution, with their animal pole facing the coverslip, for 10–20 minutes. The oocytes were then suctioned using a Pasteur pipette, leaving a giant membrane patch attached to the coverslip, with the cytosolic face toward the medium. The coverslip was washed thoroughly with fresh ND96 solution and fixated using 4% formaldehyde for 30 minutes. Fixated giant PM patches were immunostained in 5% milk in phosphate buffer solution (PBS). Non-specific binding was blocked with Donkey IgG 1:200 (Jackson ImmunoResearch, West Grove, PA, USA). Anti-Kir3.1 (GIRK1) antibody (Alomone labs, APC-005) or Anti-Kir3.3 (GIRK3) antibody (Alomone labs, APC-038) were applied at 1:200 or 1:100 dilution respectively, for 45 minutes at 37 °C. Anti-rabbit IgG DyLight650-labeled secondary antibody 1:400 (Abcam, ab96886) was then applied for 30 minutes in 37 °C, washed with PBS, and mounted on a slide for visualization. Immunostained slides were kept in 4 °C for no more than a week.

### Confocal imaging

Confocal imaging and analysis were performed as described^34,80^, with a Zeiss 510 META confocal microscope, using a 20x objective. In whole oocytes, the image was focused on oocyte’s animal (dark) hemisphere, at the equator. Images were acquired using spectral (λ)-mode: CFP was excited with a 405 nm laser and emission was collected at 481–492 nm. YFP was excited with the 514 nm line of the argon laser and emission was collected at 535–546 nm. Fluorescent signals were averaged from 3 regions of interest (ROI) at the PM and 3 similar ROIs from the coverslip outside the oocyte’s image, using Zeiss LSM Image Browser. The average background signal was subtracted from the average PM signal in each oocyte, and then the average net signal from the membrane of uninjected (naïve) oocytes was subtracted as well.

Imaging of proteins in giant PM patches was performed using the confocal microscope in λ-mode. DyLight650 was excited using 633 nm laser and emission was collected at 663–673 nm. Images centered on edges of the membrane patches, so that background fluorescence from coverslip could be seen. Two ROIs were chosen: one comprising most of the membrane patch within the field of view, and another comprising background fluorescence, which was subtracted from the signal obtained from the patch. The signal from giant PM patches of naive oocytes’ membranes, immunostained using the same protocol, was subtracted from all groups.

### Statistical analysis and data presentation

Imaging data on protein expression, as well as GIRK currents data collected from several experiments, have been normalized as described previously^87^. Fluorescence intensity or current in each oocyte was normalized to the average signal in the oocytes of the control group of the same experiment. This procedure yields average normalized intensity or current, as well statistical variability (e.g. SEM), in all treatment groups as well as in the control group. Statistical analysis was always performed on raw data with SigmaPlot 11 or SigmaPlot 13 (Systat Software Inc., San Jose, CA, USA). Two-group comparisons were performed using t-test if the data passed the Shapiro-Wilk normality test and the equal variance test, otherwise we used the Mann-Whitney Rank Sum Test. Multiple group comparisons were done using one-way ANOVA (ANOVA on ranks was performed whenever the data did not distribute normally). Tukey’s or Dunnet’s tests were performed for normally distributed data and Dunn’s test otherwise. The data in the graphs are presented as mean ± SEM or as raw data with superimposed box plots indicating 25–75 percentiles (box borders), median, mean (usually red line), and for some sets of data also 5–95 percentiles (whiskers).

## Electronic supplementary material

Supplementary Information

## Data Availability

The data that support the findings of this study are available from the corresponding author, N.D, upon reasonable request.
