# Supplementary material for: Mutual action by Gγ and Gβ for optimal activation of GIRK channels in a channel subunit-specific manner
Source: Sci Rep. 2019 Jan 24;9:508. doi: 10.1038/s41598-018-36833-y (PMC6346094; doi:10.1038/s41598-018-36833-y)
Supplement: Supplementary file 1 — Supplementary Information [file 41598_2018_36833_MOESM1_ESM.pdf]

Supplementary Information

Mutual action by Gγ and Gβ for optimal activation of GIRK channels in a channel subunit-specific manner

Galit Tabak[1], Tal Keren-Raifman[1], Uri Kahanovitch[1,2], Nathan Dascal[1,*]

[1] Department of Physiology and Pharmacology and Sagol School of Neuroscience, Sackler School of Medicine, Tel Aviv University, Tel Aviv 69978, Israel
[2] Present address: Virginia Tech School of Neuroscience, Blacksburg, VA 24061, USA

* Correspondence and requests for materials should be addressed to N.D. (email: dascaln@tauex.tau.ac.il)

Supplementary Methods (to Supplementary Fig. 3A)

[^{35}S]-methionine-labeled $G\beta_1\gamma_2$ was synthesized *in vitro* (*ivt*) in rabbit reticulocyte lysate (Promega). Interaction of His-fused proteins with [^{35}S]-Gβγ was studied by pulldown on Ni-NTA agarose beads in high K^+ buffer containing, in mM: 150 KCl, 50 Tris, 0.6 MgCl2, pH 7.0, supplemented with 0.0.5% Lubrol. The His-phosducin (5 µg) or His-SUMO (5 µg) were mixed with 5 µl of Gβγ-containing reticulocyte lysate, incubated in 50 µl for 20 min at 30°C, then the volume was brought to 300 µl and a incubated for 1 hour at room temperature (22-25°C). After removal of 5 µl from the mixture (later used as the "input" lane in SDS-polyacrylamide (12%) gel electrophoresis), 3 µl of 1 M imidazole and 30 µl of Ni-agarose beads were added to the test mixture and incubated for 30 min at 4-8°C, then washed 3 times with 500 µl of incubation buffer containing 15 mM imidazole. Elution was done by 10 min incubation at room temperature with 30 µl of the incubation buffer containing 250 mM imidazole. *Ivt*-translated lysates were added at 5 µl. The radioactive signals from protein bands of the gels were imaged using PhosphorImager and analyzed using ImageQuaNT (Molecular Dynamics). His-SUMO was a kind gift from Dr. Joel Hirsch, Tel Aviv University.

Supplementary Figures

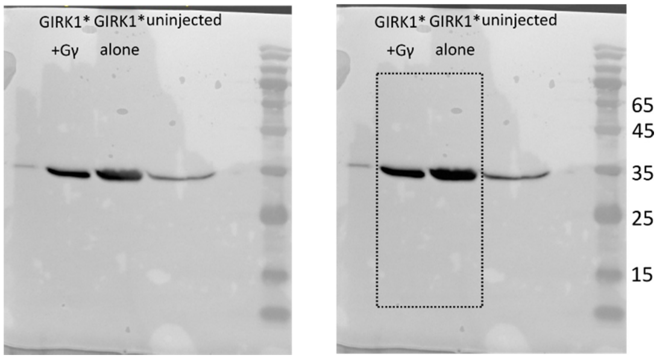

Figure S1 (to Fig. 1). Expression of Gγ does not increase the PM levels of endogenous Gβ. The complete result of the experiment shown in Fig. 1D. The figure shows two images taken with Fusion FX7 at two different exposure times (left and right). The right image was used for the production of Fig. 1D (cropped area indicated with rectangle). Western blot of manually separated PMs (25 oocytes/lane). The lane that contained PMs of uninjected oocytes is not presented in Fig. 1D because in other experiments we have not tested uninjected oocytes. The increase in the amount of Gβ in the PM following the expression of the channel may be due to the Gβγ recruitment phenomenon described in our previous publications (see main text of the paper). Oocytes were injected with 0.2 ng of GIRK1* RNA, with or without 0.2 ng Gγ RNA.

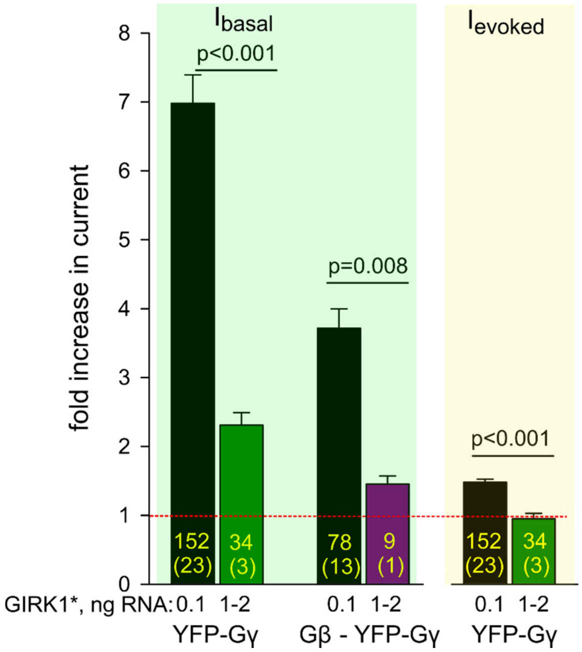

Figure S2 (to Fig. 3). At high expression levels of GIRK1*, the effects of Gγ and Gβγ are smaller than at low GIRK1* levels. Activation of GIRK1* (fold increase in I_{basal}) by YFP-Gγ or by YFP-Gβγ is milder when oocytes are injected with 1-2 ng RNA of GIRK1*, compared with the standard dose of 0.2 ng GIRK1* RNA (left and middle panels). The increase in I_{evoked} caused by coexpression by YFP-Gγ is much milder than for I_{basal}, ~1.48 fold, for the low expression level of GIRK1* (0.2 ng RNA) and it completely disappears when the channel is expressed at a higher level, 1-2 ng RNA (right panel). Number of cells (n) and N (experiments) is shown within columns. Statistical analysis: t-test on means.

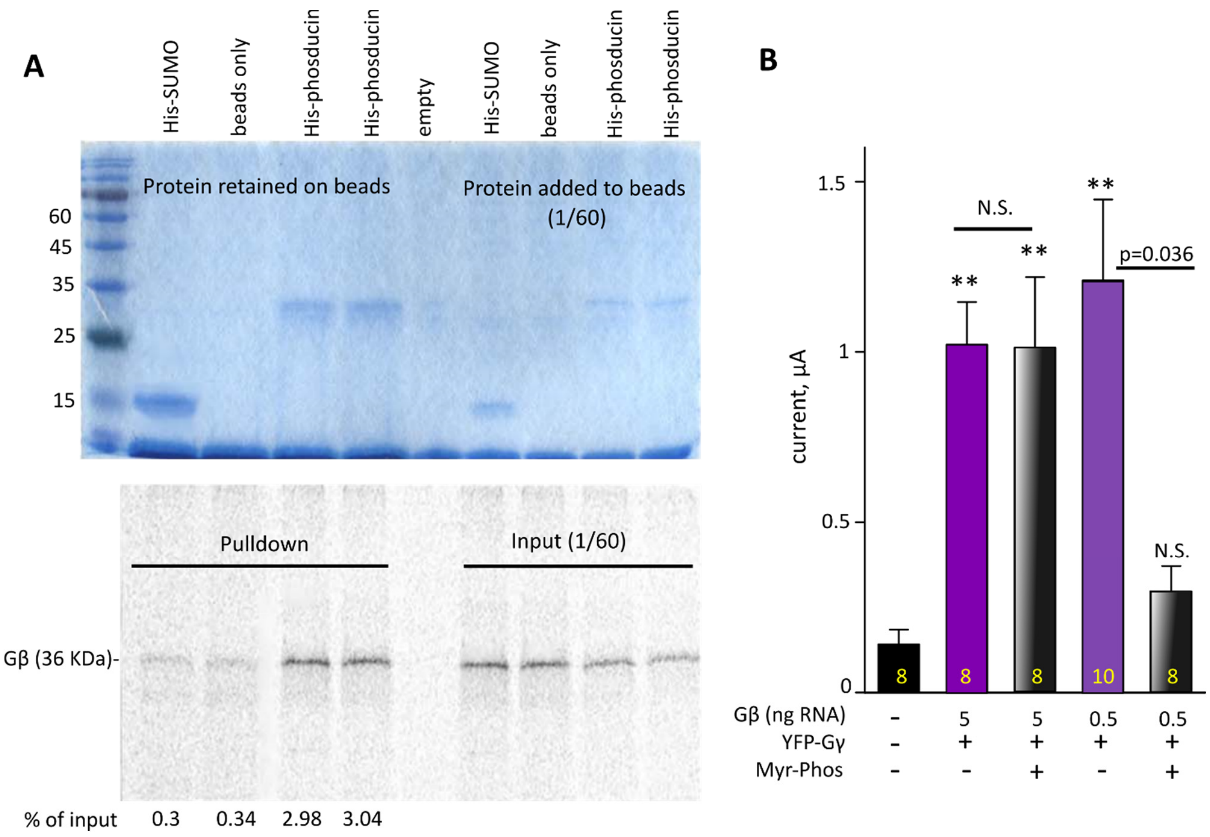

Figure S3 (to Fig. 4). Phosducin binds Gβγ, and coexpressed myr-phosducin inhibits Gβγ-induced activation of GIRK1*. A. Purified recombinant His-phosducin binds *in vitro* synthesized, [^{35}S]Met Gβγ. A representative experiment (out of 2) is shown. Gβγ was synthesized *in vitro* and pulled down by His-tagged SUMO (negative control) or Gβγ proteins on Ni-NTA affinity beads. The upper image shows Coomassie staining of the gel, and the lower image shows the autoradiogram that detects the radioactive Gβγ. The left 4 lanes show pulled-down Gβγ, the right 4 lanes correspond to 1/60 (5 μl out of total of 300 μl) of input, i.e. the total initial reaction. Normalized amount of Gβγ bound, in % of input, is shown below the bottom image. His-SUMO did not pull down Gβγ (0.3% of input, indistinguishable from background noise obtained with beads only (0.34% of input). His-phosducin pulled down Gβγ, producing a signal ~10 fold above noise (~3% of input). **B.** Coexpressed myr-phosducin inhibits GIRK1* activation by Gβγ when a low dose of Gβ is expressed (0.5 ng RNA) but not when a high does is expressed (5 ng RNA). GIRK1* (0.2 ng RNA) was present in all experiments. RNA of YFP-Gγ was 1.5 ng, myr-phosducin 5 ng per oocyte. Results of one experiment are shown; number of cells (n) is shown within columns. Statistics: raw data from all groups were compared with control (no Gβγ) **, $p<0.01$ (one-way ANOVA on ranks, Dunn's pairwise test). In addition, effect of myr-phosducin on oocytes injected with YFP-Gγ and either 0.5 or 5 ng Gβ RNA was tested by t-test (Mann-Whitney Rank Sum Test; horizontal lines above bars).

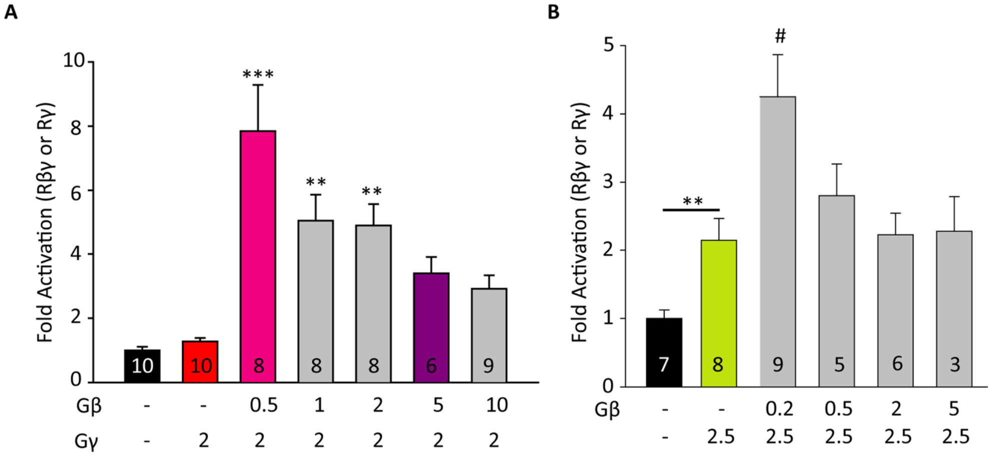

Figure S4 (to Fig. 5). Gβ enhances the activation of GIRK1* by Gγ in a complex manner. Note that Gβ best enhances the activation by Gγ at low expression levels; with higher doses of Gβ, the extent of enhancement is reduced. **A.** Oocytes were injected with GIRK1* (0.2 ng RNA) with different Gβ RNA doses (0.5-10 ng) and Gγ (2 ng RNA). The graph shows Rβγ or Rγ in each group. Data from one experiment. **, p<0.01, ***, p<0.001, relative to GIRK1* co-expressed with Gγ without Gβ (one-way ANOVA for all groups with Gγ). **B.** Oocytes were injected with GIRK1* (0.2 ng RNA) with different Gβ RNA doses (0.2-5 ng) and YFP_{A207K}-Gγ (2.5 ng RNA). Data from one experiment. #, p<0.05, relative to GIRK1* co-expressed with YFP_{A207K}-Gγ one-way ANOVA for all groups with YFP_{A207A}-Gγ); **, p<0.01 relative to GIRK1* alone (t-test).

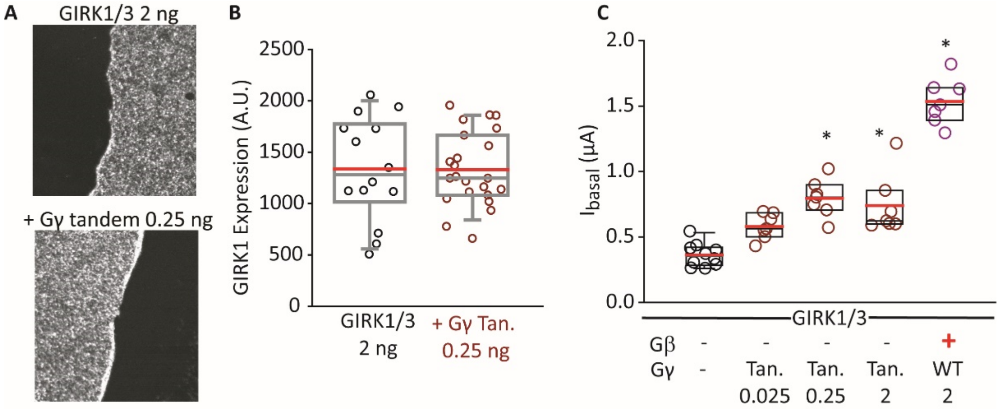

Figure S5 (to Fig. 7). Expression of Gγ tandem (Gγ Tan) increases GIRK1/3 basal activity but does not affect surface expression. A. Representative images of giant excised PM patches (GMPs) of oocytes expressing GIRK1/3 2 ng alone or with Gγ tandem (0.25 ng RNA). Oocytes were stained with an antibody against GIRK1. **B.** Summary of GIRK1/3 expression; coexpression of Gγ tandem did not significantly affect the level of the channel in the PM (t-test). n=14-23, N=1. A.U., arbitrary units. **C.** Summary of I_{basal} from the experiment in A and B. n=7-9, N=1. Gγ tandem, at 0.25 and 2 ng RNA, and Gβγ (5:2 ng RNA), significantly increase I_{basal} (one-way ANOVA, *, p<0.05 relative to GIRK1/3).

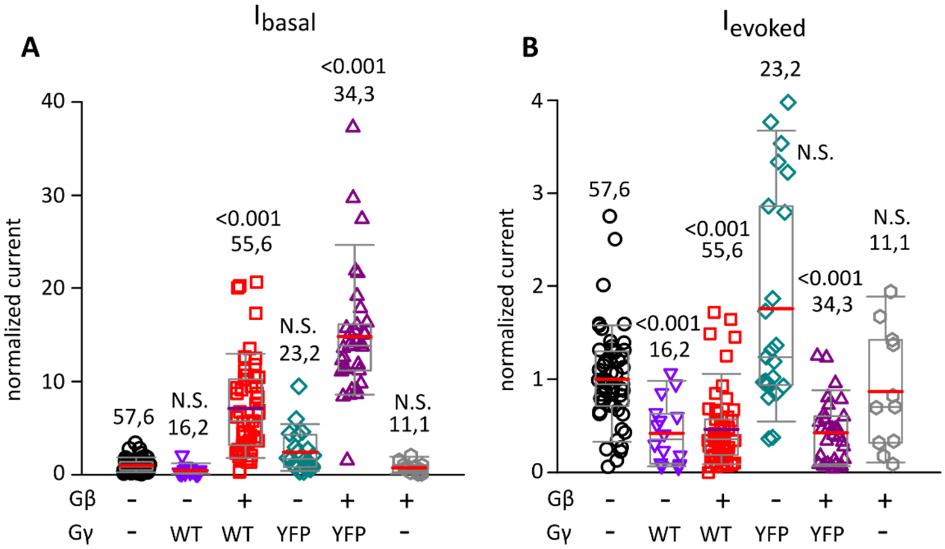

Figure S6 (to Fig. 8). GIRK1*Δ121 channel: removal of distal C-terminus of GIRK1* suppresses Gγ enhancement of I_{basal} (A) and I_{evoked} (B). RNAs were injected at the following amounts: $GIRK1_{\Delta121}$*, 2 ng; Gβ, 5 ng; Gγ, 1 or 2 ng; YFP-Gγ, 2.5 ng. Currents were normalized as follows: for each treatment, in each oocyte, including the oocytes of the control group (channel alone) the value of I_{basal} or I_{evoked} was divided by the average I_{basal} or I_{evoked}, respectively, of the control group. Numbers above data sets are n, N. Statistics: one-way ANOVA on ranks followed by Dunnet's test. Although not strictly applicable, t-test performed separately between the YFP-Gγ+ $GIRK1*_{\Delta121}$ vs. control $GIRK1*_{\Delta121}$ group showed statistically significant difference, p<0.01.
